# Harnessing Indigenous Tweets: The Reo Māori Twitter corpus

**DOI:** 10.1007/s10579-022-09580-w

**Published:** 2022-02-14

**Authors:** David Trye, Te Taka Keegan, Paora Mato, Mark Apperley

**Affiliations:** 1 School of Computing and Mathematical Sciences, University of Waikato, Hamilton, New Zealand; 2 Faculty of Māori and Indigenous Studies, University of Waikato, Hamilton, New Zealand

**Keywords:** Te reo Māori, Twitter, Social media, Corpus linguistics, Indigenous languages, Endangered languages

## Abstract

Te reo Māori, the Indigenous language of Aotearoa New Zealand, is a distinctive feature of the nation’s cultural heritage. This paper documents our efforts to build a corpus of 79,000 Māori-language tweets using computational methods. The *Reo Māori Twitter (RMT) Corpus* was created by targeting Māori-language users identified by the *Indigenous Tweets* website, pre-processing their data and filtering out non-Māori tweets, together with other sources of noise. Our motivation for creating such a resource is three-fold: (1) it serves as a rich and unique dataset for linguistic analysis of te reo Māori on social media; (2) it can be used as training data to develop and augment Natural Language Processing (NLP) tools with robust, real-world Māori-language applications; and (3) it will potentially promote awareness of, and encourage positive interaction with, the growing community of Māori tweeters, thereby increasing the use and visibility of te reo Māori in an online environment. While the corpus captures data from 2007 to 2020, our analysis shows that the number of tweets in the RMT Corpus peaked in 2014, and the number of active tweeters peaked in 2017, although at least 600 users were still active in 2020. To the best of our knowledge, the RMT Corpus is the largest publicly-available collection of social media data containing (almost) exclusively Māori text, making it a useful resource for language experts, NLP developers and Indigenous researchers alike.

**Supplementary Information:**

The online version contains supplementary material available at 10.1007/s10579-022-09580-w.

## Introduction

*Ka ngaro te reo, ka ngaro tāua*, *pērā i te ngaro o te Moa.* “If the language is lost, man will be lost, as dead as the Moa.” (Waitangi Tribunal, 1986, p. 7).

Te reo Māori^1^ (the Māori language) is intrinsic to Māori culture, and constitutes an important part of New Zealand’s national identity. In spite of this, the Māori language is currently endangered, largely due to the ongoing effects of colonial contact and the resultant marginalisation of the Māori people, their language and their culture. While significant effort has been made to revitalise the Māori language over the past 30–50 years (see King, 2018; Harlow & Barbour, 2013; Harlow, 2007), substantial work remains “to mitigate ongoing language shift and loss for Māori … in Aotearoa/New Zealand” (May & Hill, 2018, p. 317).

This paper documents efforts to compile a corpus of Māori-language tweets, which we call the *Reo Māori Twitter Corpus* (hereafter, the *RMT Corpus*). The RMT Corpus consists of 79,018 original tweets, comprising just over one million words and capturing output from 2302 accounts. It is anticipated that the creation of this resource, and any others derived from it in the future, will enhance the study, status, use and, therefore, the health of te reo Māori in wider contexts.

### Roadmap

The structure of the paper is as follows. Section 2 provides important background information and explains our motivation for this work. In Sect. 3, we describe related work, focusing on existing Māori corpora and Natural Language Processing (NLP) resources, as well as highlighting issues of data sovereignty from an Indigenous Māori perspective. Section 4 details the method for building the RMT Corpus, summarising the process in four main steps. A preliminary analysis of the RMT Corpus, which we plan to extend in future work, is given in Sect. 5. Section 6 explains how researchers can download the RMT Corpus and associated resources. Finally, in Sect. 7, we present conclusions and suggestions for future work.

## Motivation

Modern NLP resources are usually developed using quantitative methods that rely on the existence of large corpora. Unfortunately, substantial corpora for minority languages (such as Māori) are scarce; they exist mostly for languages that have a market-driven need for NLP tools, such as English, French, German and Cantonese. In fact, it has been estimated that significant digital resources exist for only 20–30 of the world’s 6000 + languages (Maxwell & Hughes, 2006, as cited in Bloem et al., 2019; see also Scannell, 2007). As an endangered language spoken by roughly only four per cent of the New Zealand population,^2^ it is not surprising that Māori falls outside this category. A lack of appropriate corpora hampers the development of critical NLP resources for the Māori language (James et al., 2020). We suggest that a corpus of Māori tweets—while no panacea—is a step in the right direction for future progress in NLP for te reo Māori.

The internet and the proliferation of social media provide unprecedented access to many low-resource languages. Large amounts of untapped, real-time data are available on popular social media sites, such as Twitter, Facebook and Instagram, creating an ideal hunting ground for computational linguists (King, 2015). Anecdotal evidence suggests that the Māori language is increasingly being used and discussed on these platforms, as greater numbers of New Zealanders endeavour to incorporate te reo Māori in their everyday lives. Such outcomes are the fruit of a wide range of language revitalisation initiatives aimed at promoting the use of te reo Māori in everyday contexts. For instance, a national social media campaign was launched by Te Māngai Pāho (the Māori Broadcast Funding Agency) in September 2019 to achieve a target of one million te reo Māori tweets by New Zealanders, using the hashtags *#MahuruMaori*, *#1MirionaTihau* and *#1MirionaTweets*.^3^

Corpora derived from social media have a diverse range of applications. They can be used to build language models, train word embeddings and perform various kinds of machine learning and NLP tasks, such as part-of-speech tagging, morphological analysis, parsing, information retrieval, speech recognition and machine translation (see, for example, El-haj et al., 2015). This is particularly important for languages such as Māori, because the creation of new NLP resources for endangered languages also enhances their profile, contributing to more positive attitudes towards the language, and a sense of greater relevance and modernity. This in turn attracts new learners (Coto Solano et al., 2018) and increases language prestige (Bender, 2019; Meyerhoff, 2019).

Social media corpora are also gaining prominence in linguistics. In the context of minority languages, these corpora are especially useful when limited data are available elsewhere, or when existing resources suffer from a lack of currency and diversity. Twitter provides a source of natural, user-generated content that is very different from other, more traditional genres. Cassels (2019) notes that social media sites are conducive to spontaneous literacy production and non-prescriptivist language use, reflective of everyday usage. As a result, linguistic phenomena rarely seen in formal settings are more likely to be present on Twitter (Lynn & Scannell, 2019). Moreover, since tweets often resemble spoken language more closely than formal written language, they are more likely to be suitably used as training data for text-to-speech and speech-to-text applications.

Research also indicates that social media and the internet can play an important role in supporting the revitalisation of minority languages. This is in keeping with traditional language revitalisation theory (see, for example, Giles et al., 1977 and Zuckermann, 2020), which maintains that the more domains in which a language is used, the stronger its *ethnolinguistic vitality*. Languages with strong ethnolinguistic vitality are visible and accepted within their communities and, consequently, more likely to be transmitted to future generations (Meyerhoff, 2019). Cunliffe et al., (2013, p. 75) found that social media sites help to establish “linguistic communities” and “revive weakened languages”. In a similar vein, Ka‘ai (2017, p. 30) observes that “minority-language users can, through the internet and new media, become producers as well as consumers of media products in their own language” (see also Ka‘ai et al., 2012). This is not to say that the use of a minority language on social media and the internet will *prevent* its extinction; there are many other important contributing factors, such as the number of speakers, the level of institutional support that the language receives and the strength of intergenerational transmission (Bird, 2020). However, young people’s importance in language survival and their increasing comfort and dependence on social media communications cannot be overlooked (Keegan & Cunliffe, 2014).

From a Māori perspective, there are a range of benefits and drawbacks concerning the uptake of social media. Sciascia (2016, p. 6) notes that social media sites can function as a “virtual *marae*” [gathering space] where the Māori language is “becoming a normalised form of communication” and “*tikanga* [customs/values] are being practiced”. In particular, social media aligns with the Māori values of *tino rangatiratanga* (self-determination) and *whanaungatanga* (relationships/networks). The term *e-whanaungatanga* (electronic relationships/networks) was coined in recognition of the implications of social media for tikanga Māori, to capture the notion of cultivating positive relationships online (Waitoa et al., 2015).

As regards disadvantages, social media increases susceptibility to cultural misappropriation and can be used as a platform for targeting ethnic minorities and/or spreading racist views and disinformation—all of which clearly oppose Māori values. Furthermore, the widespread use of artificial intelligence (AI) on digital platforms poses ethical concerns relating to algorithmic bias, discrimination, privacy (Bender et al., 2020) and especially data sovereignty (Whaanga, 2020; see Sect. 3.2). Another drawback of the use of social media is the impact of diaspora and the fact that Māori are less inclined to travel back to their *tūrangawaewae* (domicile of origin) because they are connected through social media. However, at the same time, social media does enable the displaced or diasporic to continue using their language, and to maintain their connections to their homelands and each other.

The benefits and drawbacks of the use of te reo Māori on social media platforms are part of a much larger discussion; however, we believe there is significant value in creating a corpus of Māori-language tweets. Twitter has been selected in favour of other social media platforms, due to the unidirectional nature of its network and the accessibility of data through the Twitter API. For the most part, Twitter users are able to follow other users without those users’ permission. The same is true for developers wanting to download their tweets. We are confident, therefore, that Māori-language data can be more readily extracted from Twitter than from other sites such as Facebook, whose privacy settings severely limit the amount of data that can be collected. Targeting the Twitter platform enables the creation of a larger corpus than we might get from other platforms. Additionally, the aforementioned Te Māngai Pāho campaign was specific to Twitter, and that significant amount of generated data is not available elsewhere.

The RMT Corpus lends itself to linguistic analysis of informal Māori language as it captures authentic, user-generated content from real (human) tweeters of te reo. Furthermore, the corpus offers great potential for contributing to the development of new and important NLP resources for the Māori-language community. For instance, it could be used as a golden set (benchmark) of Māori-annotated tweets for language classification purposes, as training data for text-to-speech and speech-to-text applications, or as dictionary input for a reo Māori auto-completion tool (Innes, 2021). To this end, the corpus has already been shared with an iwi media organisation to support their language revitalisation efforts, as well as several research institutes across New Zealand. Through the networks of the Māori authors of this paper, the corpus will also be shared with Māori-language teachers who are interested in using real language examples and creating fresh learning materials for their students.

## Related work

The following is a summary of the resources and techniques that informed our approach to building a corpus of Māori-language tweets.

The present study builds on the findings of two prior investigations into Māori language use on Twitter. Mato and Keegan (2013) analysed the ten most prolific users of te reo Māori and found that they tweeted primarily to disseminate religious text, news items or organisational notices, rather than to present their own thoughts and opinions, or engage interactively with other users (p. 190). In a follow-up study, Keegan et al. (2015) found that a small yet dedicated group of individuals on Twitter was actively conversing in te reo Māori, especially during events with strong cultural significance, such as Māori Language Week^4^ and Te Matatini, a nation-wide Māori performing arts festival and competition. This paper serves as an update to these studies, whilst also collating a Māori-language corpus: a first-of-a-kind resource made available to other researchers. A defining feature of the RMT Corpus is the enriched metadata associated with both the tweets and their authors, which affords new possibilities for exploring Māori-language use on social media, especially in terms of content and location (however, such exploration is beyond the scope of the present study).

### Existing Māori-language resources

Several Māori-language corpora have been compiled over the past few decades. These resources typically capture more formal language than one would expect to see on Twitter, arising from genres such as legal texts and speeches, television broadcasts and children’s literature. Table 1 provides a summary of each corpus, citing information about their size, genre and availability.

**Table 1:** Summary of existing Māori-language corpora

Title	Date	Description	Medium	Publicly available	Source/citation
Legal Māori Corpus (LMC)	1829–2009	8 million words from legal and law-related texts; the largest structured corpus of the Māori language	Written	Partially: Only data from before 1910 (5.3 million words) can be downloaded, but the entire corpus is searchable online^a^	Boyce (2011)
Niupepa Collection	1842–1932	Over 17,000 pages of historic newspaper text, based on a microfiche collection produced by the Alexander Turnbull Library. 70% of the collection is written exclusively in Māori	Written	Yes^b^	Apperley et al. (2002)
Hansard Corpus	1860–2018	573,000 words from NZ Parliament utterances that are predominantly in Māori	Spoken	Yes^c^	Te Hiku Media
MAONZE Corpus	Late 1940s; 2001–2009	Transcribed recordings of the Māori and English speech of three generations of Māori (~ 109 h, 620,700 words)	Spoken	No	King et al. (2010)
Tūhoe Corpus	2009	Transcribed recordings of five male and five female elders of the Tūhoe tribe (~ 19 h, 114,000 words)	Spoken	No	King et al. (2010)
Māori Broadcast Corpus (MBC)	1995–1996	Transcripts of one million words of radio and television broadcasts	Spoken	No	Boyce (2006)
Māori Texts for Children (MTC)	Unknown	A collection of Māori texts for children	Written	No	Huia Publishers
Te Paipera Tapu	2012 (originally translated in 1868)	A one-million word Māori translation of the Bible, reformatted to include macrons	Written	Yes: Users can download the *Te Paipera Tapu* app, or view the text online^d^	New Zealand Bible Society
Māori Written Corpus	2009–2012	3.7 million words of Māori text, sourced from personal collections, language friends, public and government sites (personal communication, February 24, 2021)	Written	Yes: On request	Te Taka Keegan
Māori Speech Corpus	2019–2020	A Māori speech corpus, sourced from *Ngā Mahi a Ngā tūpuna* (Grey, 1928), together with a lexicon of 10,000 words and 18,000 names, and 1030 recorded sentences	Spoken	No	James et al. (2020)
Papa Reo Dataset	2017–2020	A dataset compiled by Te Hiku Media, based on 198,000 speech recordings from 2200 speakers, comprising 4.8 million words and 5000 unique sentences	Spoken	No, but see the resulting tools in footnote 11	Moses et al. (2020)

^a^See http://nzetc.victoria.ac.nz/tm/scholarly/tei-legalMaoriCorpus.html and https://www.legalmaori.net/corpus

^b^See http://www.nzdl.org/cgi-bin/library.cgi?a=p&p=about&c=niupepa

^c^See https://github.com/TeHikuMedia/nga-tautohetohe-reo

^d^See https://biblesociety.org.nz/discover-the-bible/the-bible-in-maori/maori-bible-app/ and https://www.biblegateway.com/versions/Maori-Bible/#booklist

The RMT Corpus complements this existing body of Māori-language text since, although a mixed-language corpus of three million English tweets exists that contains borrowed Māori words or loanwords (Trye et al., 2019, 2020), there is, to the best of our knowledge, no social media corpus comprising (almost) exclusively Māori-language text.

Te reo Māori has more recently received considerable attention from the NLP community (more so than other Polynesian languages; see Coto Solano et al., 2018). For instance, te reo Māori is supported by the popular machine translation tool *Google Translate,*^5^ an online tool exists for automatically restoring Māori macrons,^6^ and SwiftKey has developed a custom Māori keyboard. Other initiatives have yielded the first automatic Māori speech recogniser and transcription tool, recently developed by Te Hiku Media,^7^ and the use of deep learning methods to provide instant, character-by-character pronunciation feedback to learners of te reo Māori (Moses et al., 2020). In a related project, a synthetic male voice was developed, allowing synthesised speech to be generated from any Māori text input (Shields et al., 2019). Furthermore, Te Hiku Media is in the process of developing the first reo Māori part-of-speech (POS) tagger (Finn, 2021). Despite these and other similar developments, advancements for te reo Māori, in terms of the data and equipment that are required to build robust speech technologies and language models, remain woefully under-resourced (James et al., 2020).

### Data sovereignty

Indigenous communities are becoming increasingly aware of the importance of maintaining control and sovereignty over their data, and of the potential repercussions of not being able to do so (Kukutai & Taylor, 2016). Te Hiku Media advocates a moral responsibility to “respect the *mana* [honour/power/respect] of the data and the people from whom it descends”.^8^ Unfortunately, this is not always straightforward. In today’s culture of open data and open science, the sharing of information can perpetuate the misuse and subsequent exploitation of data for commercial gain—more especially by large corporations (even if this is not the intention of the people who collect and share that data). Even so, Indigenous people can benefit hugely from cutting-edge contemporary technologies, particularly in terms of currency and scalability. However, they can also be exposed to further marginalisation and data appropriation; a form of modern colonisation in the digital space (Keegan et al., 2021). Bird (2020) calls for speech and language technologists to consider their own role in commodifying Indigenous knowledge as data, cautioning against techno-solutionism in NLP. In terms of this research, we believe it is appropriate to make the RMT Corpus widely available as a resource that has been sourced from an open platform, which is intended to be used by academics and Indigenous researchers to benefit Māori and the Māori-language community.^9^

### Welsh Twitter corpus

While there have been several exciting developments in the world of Māori NLP (see Sect. 3.1), none of these resources makes use of the rich and ever-increasing amount of data available on social media. The *Welsh Twitter Corpus* (Jones et al., 2015) demonstrates the advantages of building a Twitter corpus for a minority language. Developed at Bangor University, the corpus comprises seven million Welsh-language tweets, and helps to meet “great demand” for informal Welsh data.^10^ The authors identify several potential applications for the *Welsh Twitter Corpus*, which we believe are also relevant to the RMT Corpus in the context of te reo:

- Training predictive text systems for [sms on] phones
- Finding new words in the Welsh [language]
- Developing research material for academic departments in Universities, including in linguistic, sociological and medical studies
- Educational and demonstration data for children and young people in coding clubs
- Valuable information for the market, for example; tracking and analysing user emotions (sentiment analysis).^11^

## Building the RMT Corpus

In this section, we explain our methodology for building the RMT Corpus. Our main objective was to create a corpus of (almost) exclusively Māori-language tweets.

Initially, we tried to mirror the approach employed by Trye et al. (2019), which used a seed list of Māori *query words* to build a corpus of New Zealand English tweets (English tweets containing Māori borrowings). To this end, we compiled a set of Māori words and phrases that we considered would be useful for harvesting tweets explicitly tagged as Māori. However, it soon became clear that this approach would not work as intended, because Twitter does not provide official support for te reo Māori. This meant that we could not automatically extract Māori-language tweets using only the Twitter API.

Instead, we decided to gather (and clean) data from known *users* of te reo. Targeting a set of relevant users is a well-established method for compiling a Twitter corpus (see, for instance, Verhoeven et al., 2016 and Zaghouani & Charfi, 2018). Māori-language tweeters were identified using the *Indigenous Tweets*^12^ website, which periodically crawls the web for tweets written in 185 minority languages. Since our approach relies heavily on *Indigenous Tweets*, we provide a brief overview of its functionality in Sect. 4.1, and describe relevant limitations in Sect. 4.2.

**Fig. 1:**
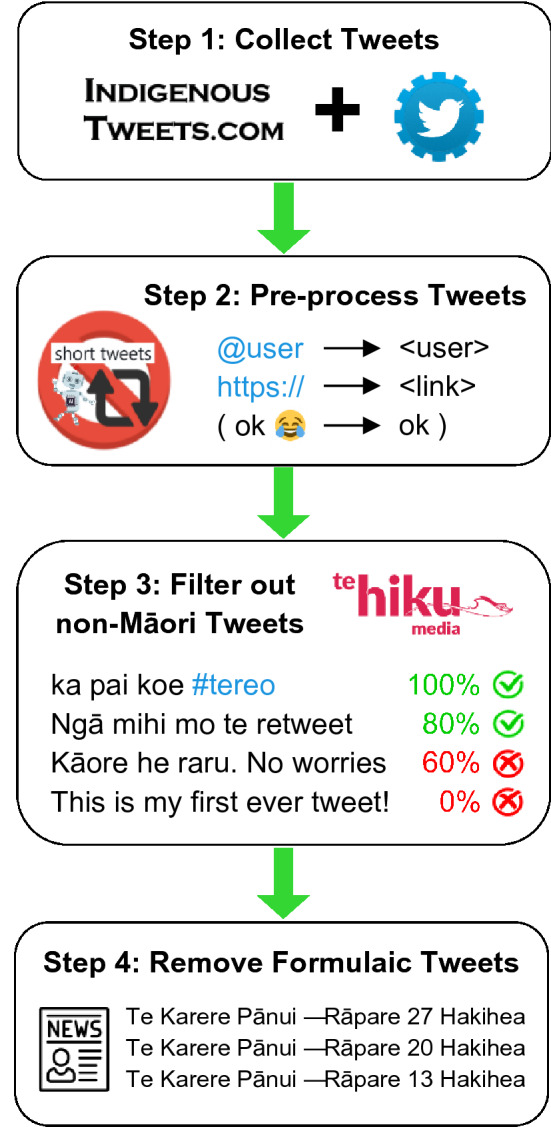
The process of building the RMT Corpus, broken down into four key steps

**Fig. 2:**
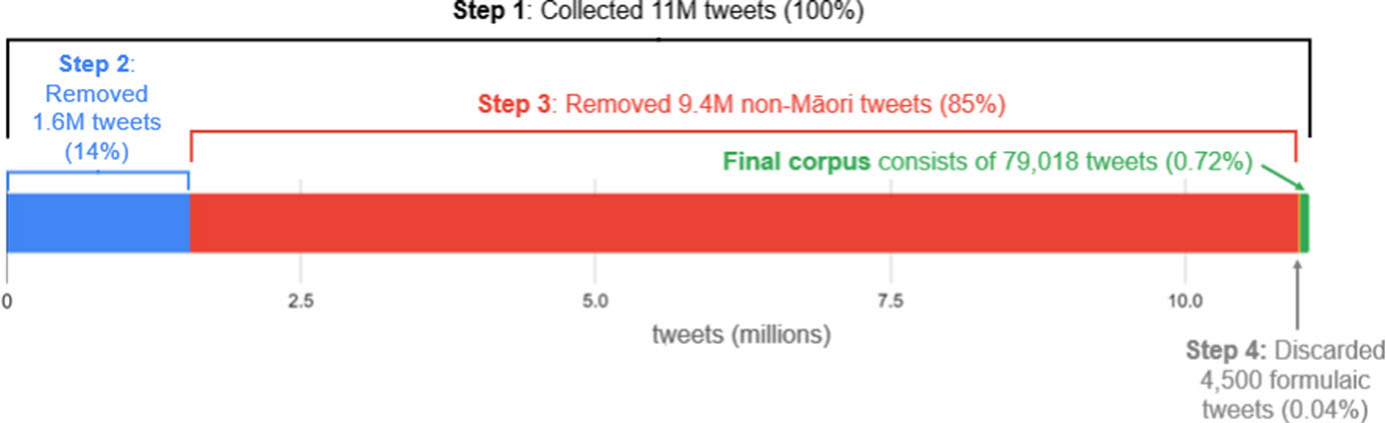
Visualisation showing the proportion of tweets removed during each step. The width of the entire bar represents the 11 million tweets that were initially collected, and the green bar on the right represents the final corpus of 79,018 tweets. The vast majority of tweets (85%) were removed during Step 3, after calculating the percentage of Māori text in each tweet. (Color figure online)

We now provide an overview of how the RMT Corpus was created, before explaining each step in detail. The corpus-building process can be summarised in four main steps, as shown in Figs. 1 and 2. In step one, the Twitter API^13^ was used to download 11 million tweets from Māori users listed on the *Indigenous Tweets* website (Sect. 4.2). Because users tend to post much more frequently in English than Māori, only a small portion of these tweets were written in te reo. Step two involved pre-processing the data to enhance readability and mitigate noise in the corpus. At this point, 1.5 million short tweets were discarded, along with 60,000 tweets from automated accounts. Tweet text was also formatted such that *@user* mentions and hyperlinks were standardised. Two distinct versions of the text were extracted: one in which special characters such as emojis were preserved, and the other in which these were eliminated (Sect. 4.3). In step three, the percentage of Māori text in each tweet was calculated, so that tweets containing less than 70–80% Māori text could be systematically removed. 9.4 million non-Māori tweets were filtered out accordingly. This step—crucial for cleaning the corpus—was carried out by leveraging NLP code developed by Te Hiku Media (Sect. 4.4). Finally, step four involved removing 4473 formulaic Māori tweets that were thought to adversely affect corpus quality (Sect. 4.5). The remaining tweets in the corpus are believed to contain original and authentic Māori text, suitable for a range of computational and linguistic tasks, as described in Sect. 2. It is worth noting that even the discarded tweets can be seen as a valuable resource, because they represent varying degrees of Māori/English code-switching (captured by the *percent_maori* attribute; Sect. 4.4).

### Indigenous Tweets background

The *Indigenous Tweets* website catalogues users of 185 Indigenous languages (as of 8 February, 2021), including te reo Māori. Created by Professor Kevin Scannell of Saint Louis University in March 2011, the website aims to connect minority-language speakers through the internet, making their voices visible in the face of language giants such as English and Spanish.^14^*Indigenous Tweets* uses a combination of character tri-grams and word features to determine whether a given tweet belongs to a particular language in its database. A separate crawler exists for each language, which scans the corresponding list of users and keeps all tweets detected in that language. The database is continually updated to not only incorporate new tweets from known users, but to also capture other (previously unidentified) users of the target language, including their older tweets. More specifically, there are four main techniques for adding tweets from new (Māori) users (for further details, see Scannell in press):

1. Leveraging distinctive (Māori) words not present in other languages;
2. Searching accounts that known (Māori) users have retweeted;
3. Querying known (Māori) users’ followers;
4. Crowdsourcing suggestions for (Māori) users via the *Indigenous Tweets* website.

The *Indigenous Tweets* home page reports key statistics for all 185 featured languages, including the total number of tweets, distinct users, the most frequent user and the user who posted the first recorded tweet for each language. When sorted by number of tweets, Māori is currently ranked sixteenth overall, with 313,122 tweets in total.^15^ Interestingly, however, Māori rises to fifth place when sorted by number of users, with what appears to be a healthy community of 3,090 users. This would suggest that, compared to other Indigenous languages with a large presence on Twitter, Māori has *more users* who post *fewer tweets.* These numbers have increased significantly over the past six years; in 2015, there were only 93,283 Māori tweets posted by 345 tweeters (Keegan et al., 2015).

**Fig. 3:**
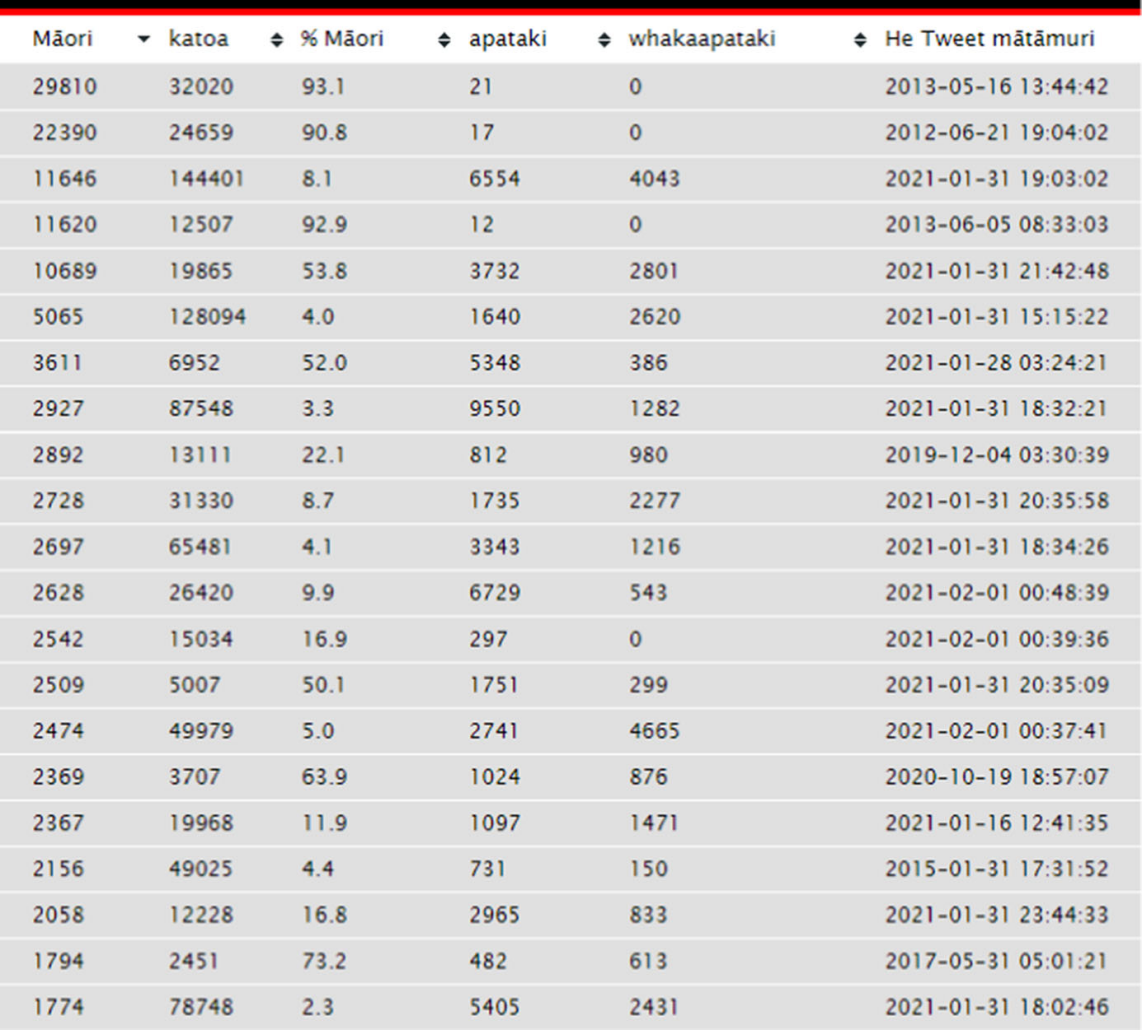
The top 20 Māori tweeters on the *Indigenous Tweets* website as of 8 February, 2021. Usernames have been omitted to protect users’ identities

Users of *Indigenous Tweets* can also view more detailed information about each language’s top 500 tweeters. Figure 3 shows the top 20 Māori tweeters, three-quarters of whom were active within the past year. Most Māori-language users only tweet in te reo a small proportion of the time, presumably tweeting mainly in English the rest of the time.^16^ These figures also include retweets, a re-posting of a tweet, which can lead to overestimates of how many tweets have been written in a particular language, although this still provides evidence of people engaging with the content in that language.

The *Indigenous Tweets* website has three notable limitations that users intending to build a minority-language corpus should be mindful of. First, the web crawlers used are not perfect and occasionally admit false positives. We found a small number of non-Māori language users whose (Indonesian and Samoan) tweets were erroneously included in the database. Second, the process of updating the list of tweeters for a given language is not entirely automated, which means the website may be missing information for recently-discovered users. Finally, the classifier does not currently support detection of multiple languages within the same tweet (e.g. due to code-switching). Assigning exactly one label to each tweet is not always representative of actual language use and may preclude bilingual users who frequently post content in two or more languages from being identified.

### Step one: collecting tweets

**Fig. 4:**
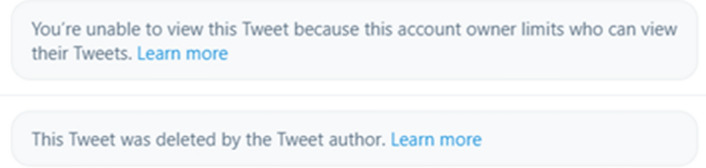
Searching for a tweet that is not publicly available on Twitter. The API returns a “Not Found Error” if a tweet has been deleted, or an “Authorization Error” if the tweet is from a protected account

As explained previously, the first step involved in creating the RMT Corpus was to collect tweets from known Māori-language users. Since the *Indigenous Tweets* website displays only the top 500 users for each language, we requested access to the usernames of all 3090 (potential) Māori tweeters (Kevin Scannell, personal communication, 21 January, 2020). The Twitter API was then used to gather as many tweets as possible from these users (including their non-Māori-language tweets). A total of 10,870,247 tweets were collected. Our search timeframe spanned a period of nearly 15 years, from Twitter’s inception in March 2006 through to December 2020, although the first Māori tweet did not occur until May 2007. Not all tweets were available for every user, due to the corresponding accounts becoming protected (accessible only to the user and their followers), or having been suspended^17^ or deleted (see Fig. 4). Moreover, some users may have changed their usernames from those specified on the *Indigenous Tweets* website, resulting in a false association. In particular, we were not able to gather data for 286 of the 3,090 listed users in 2020 (9.26%), which can also be attributed to a change in how the tweets were collected.^18^ We have augmented the tweets with a rich array of metadata (some sourced directly from the Twitter API, others manually derived), which we believe may provide useful insights into patterns of Māori-language use on Twitter.

#### Tweet metadata

The RMT Corpus has a wealth of metadata available, with (up to) 48 variables pertaining to both the tweets and their authors. This includes a mixture of *upstream metadata*, sourced directly from the Twitter API, and *derived metadata*, which we added ourselves. Unfortunately, many records are incomplete; see Sect. 4.2.3.

A selection of tweet metadata included in the corpus is detailed below (see Online Resource 1 for a description of all 48 variables):

- The text contained in the tweet, formatted as per Sect. 4.3. Users can choose whichever version of the text is best suited to their specific needs: the *content* without special characters, or the *content_with_emojis,* which includes special characters [derived].
- The tweet *ID*, which uniquely identifies the tweet and allows it to be viewed in context [upstream].
- The timestamp (*date*) when the tweet was posted, which can be used for diachronic analysis [upstream].
- The *conversation ID,* which is the ID of whichever tweet initiated the conversation^19^ [upstream].
- The number of *likes, retweets* and *quotes* associated with the tweet; so-called “public metrics” [upstream]. These three values were added together to compute the number of *favourites* [derived].
- The number of *replies* that the tweet received [upstream].
- The *language* by which the tweet was classified. Because the Twitter API does not support te reo Māori, most tweets in the corpus have been erroneously classified as another language [upstream].
- The *source* from which the tweet was posted, including a wide range of devices and third-party applications [upstream]
- *Error* information explaining why some tweet data could not be retrieved (e.g. not found or unauthorised) [upstream]
- The list of *Māori words* detected in the tweet (during Step 3; see Sect. 4.4) [derived].
- The *percentage* of Māori text detected in the tweet (during Step 3; see Sect. 4.4) [derived].

#### User metadata

Each tweet is authored by a *user* who has their own set of attributes. The RMT Corpus comprises (up to) 26 attributes for 2,302 distinct users. This information is shared among all tweets by the same author, and can be represented in a nested data structure (embedded within the tweet metadata). The author metadata is time-dependent, providing an accurate snapshot from December 2020.

A selection of user attributes included in the corpus is given below (see Online Resource 1 for the complete list):

- An *alias* for the user who posted the tweet. This is in the form *TX* , where  *X* is the user’s ranking based on their total number of tweets in the corpus, such that user *T1* is the most prolific Māori-language tweeter. We have anonymised usernames in this way to safeguard against malicious use of the data [derived].
- The account’s *status* as of April 2021 (active, protected, not found or suspended), which influences whether the tweet can (still) be downloaded via the Twitter API [upstream].
- The user’s total number of *tweets* in the corpus [derived].
- The user's *location*, based on self-reported information. Where possible, this was aggregated into New Zealand regions (e.g. “Auckland”, “Waikato”) and names of overseas countries (e.g. “France”, “Australia”). Many users did not specify their location, or did so at a higher granularity (e.g. “North Island”), which meant we could not infer the particular region where they live [derived].
- The date the user’s account was *created,* which may be fruitfully compared with the date of the user’s first Māori tweet in the corpus [upstream].
- The user’s total number of *statuses,* including non-Māori tweets and retweets [upstream].
- The user’s total number of *favourites,* calculated as the sum of all their likes, retweets and quoted tweets [upstream].
- The user’s total number of *followers* [upstream]*.*
- The number of accounts that the user follows (*friends*) [upstream].
- Whether or not the user’s account is *verified* (this applies to accounts of public interest that are “authentic, notable, and active”^20^) [upstream].

With a view to understanding more about user demographics, one of the authors of the paper coded additional information about each tweeter’s gender and ethnicity. The tagging process involved manually reading each user’s account description to see if they mentioned their ethnicity (e.g. “Māori”) and/or personal pronouns (e.g. “she/her/ia”). In some cases, these were not explicitly stated but revealed implicitly. For instance, the coder inferred that users were Māori if they mentioned their iwi/tribes, and similarly deduced their gender if they used nouns such as *māmā* or “mother” (e.g. “proud māmā of two”). Accounts representing multiple people (e.g. businesses or organisations) were labelled as “groups”. We were able to extract gender information for 85% of users (n = 1957), among whom 50% were female, 31% were male, 19% were group accounts and 0.3% were gender-neutral. This information reveals a richer picture of the social profile of Māori-language tweeters, which we intend to explore in future work.

#### Overview of missing metadata

**Fig. 5:**
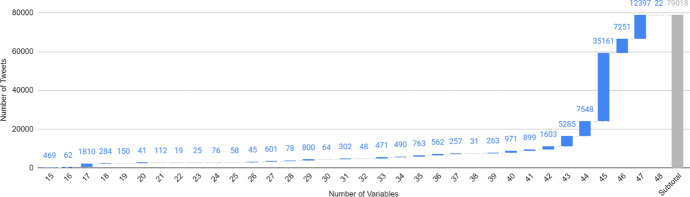
Waterfall chart showing how many tweets have data for the given number of variables. 85.63% of tweets have at least 43 of the 48 variables

While we attempted to collect data for 48 variables, most tweets are missing information for one or more of these fields. Figure 5 is a waterfall chart showing how many tweets have data recorded for the corresponding number of variables. All tweets comprise a minimum of 15 variables, including the tweet content, tweet ID, user alias, list of Māori words and percentage of Māori text. Only 11,354 tweets in the corpus (14.37%) are missing data for more than five of the 48 variables; the remaining 67,664 tweets constitute complete or almost complete records.

**Fig. 6:**
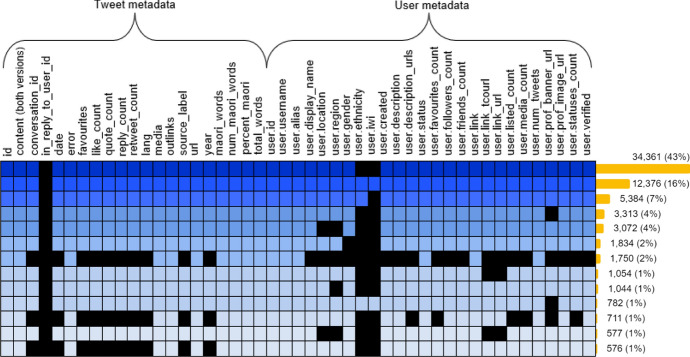
Matrix showing the most frequent *combinations* (rows) of tweet and user *metadata* (columns) in the RMT Corpus. Missing values are denoted using black cells. Drawn using Microsoft Word

Figure 6 provides a more detailed visual summary of the missing information. Specifically, the rows in the matrix show all combinations of variables that occur more than 500 times, accounting for 84% of the corpus data. The most frequent combinations are listed at the top, reinforced by the shade of the (non-black) cells and the bar chart on the right-hand side. The columns provide an indication of how many tweets have metadata for the corresponding variable, with *in_reply_to_user_id, user.iwi* and *user.ethnicity* being the most sparse variables. Note that, in some cases, there is a distinction between blank (null) values and missing (unknown) values. For example, regarding the *user.description* variable, this would be the difference between a user who does not have an account description, and a user whose account description is not known, because we were not able to retrieve it. Values in the former category are not considered to be missing.

#### Corpus collection caveats

The RMT Corpus may not be fully representative of Māori language use on Twitter. As noted at the beginning of Sect. 4.2, various constraints imposed by Twitter and our data collection method prevented us from accessing a (potentially) large number of tweets posted by certain users, especially throughout 2020. In addition, the *Indigenous Tweets* database does not provide an exhaustive list of Māori tweeters (see Scannell in press), which means we cannot claim to have accounted for every single te reo Māori tweeter. Consequently, the RMT Corpus should be regarded as capturing only a subset of relevant tweets from a subset of relevant users.

In the interests of transparency and replicability, we describe some further caveats below. Twitter’s terms of service do not allow third parties to redistribute tweet content directly: only IDs and selected metadata can be publicly shared. Anyone interested in accessing the corpus text is thus required to download (‘hydrate’) the tweets using the IDs provided, but this relies on their still being available via the Twitter API (Sect. 6). As of April 2021, 6,443 tweets in the RMT Corpus (8.15%) could not be hydrated, either because the user’s account was protected, suspended or deleted, or because individual tweets had been deleted from active accounts. Other tweets that are currently available may also become inaccessible in the future, exacerbating this problem (see further discussion in McCreadie et al., 2012).

Two key features of Twitter are the ability to reply to tweets and to package multiple related tweets in a conversation called a “thread”. Therefore, because tweets are often part of larger conversations, important context may be missing when a tweet is read in isolation. While a *conversation_id* is available for most tweets in the RMT Corpus, enabling an analyst to group tweets by conversation, there is no guarantee that every tweet in a conversation will feature in the corpus. Not all tweets belonging to a given conversation will necessarily also be written in Māori, or contain sufficient Māori text. In addition, some tweets in a conversation may not be publicly available, harking back to the previous problem.

As explained in Sect. 4.2.3, some tweets are missing a considerable amount of metadata (see Figs. 5 and 6). We assume that any self-reported user information (such as location and gender) is accurate. From a sociolinguistic perspective, the RMT Corpus was not designed to yield a balanced sample of users (in terms of variables such as region, gender and ethnicity), which affects how it can be used. Finally, analysts should be aware that each account in the RMT Corpus does not necessarily represent a discrete user, as a single person could manage multiple accounts (e.g. personal and professional accounts). However, we suspect this is unlikely to be the case for most users in the corpus.

### Step two: pre-processing the corpus

Having gathered nearly 11 million tweets from known users of te reo Māori, the next step was to clean the data and remove any tweets that would hinder analysis of the RMT Corpus. There were several major sources of noise, including tweets containing little or no Māori text, tweets containing a strong mixture of two or more languages (e.g. English/Māori code-switching), retweets and short tweets lacking sufficient context or meaning. We decided to deal with the non-language-related sources of noise first: those which could be addressed without knowing how much Māori text was in the tweet.

Several adjustments were made to enhance the quality of the corpus. We discarded retweets (re-postings of tweets) because we were not interested in capturing data from *passive* users of te reo, who shared other people’s Māori-language tweets without actively writing their own. This also ensured the removal of duplicate tweets that would have negatively skewed the data. 1,522,221 tweets containing fewer than four tokens (words) were also removed, on the basis that they carried minimal linguistic value, even if written completely in Māori (e.g. *Āe!*! meaning “Yes!”).

We then applied minor stylistic changes to the formatting of each tweet. In order to improve readability, HTML entities were decoded (e.g. *&quot;* and *&lt;*, representing a quotation mark (“) and the less-than sign (<), respectively). These characters were considered to be distracting and unhelpful. The text in the *content_with_emojis* variable preserves special characters (which are useful for tasks such as sentiment analysis), whereas the *content* variable contains only alpha-numeric characters, basic punctuation and vowels with macrons.

Adjustments were also made to textual features with special properties, such as *@user* mentions and hyperlinks. In a bid to protect users’ identities (following ethics guidelines proposed by Wilkinson and Thelwall, 2011), we replaced all *@user* mentions with “<user>”. There are 60,416 “<user>” references in the final corpus, distributed among 40,484 tweets (some tweets mention multiple users). Therefore, just over half of all tweets in the corpus include one or more user mentions. We did not want to completely erase *@user* mentions because they are sometimes integrated into the tweet text itself and can therefore contribute to the syntax/meaning of a tweet. Moreover, when we eventually deployed the rule-based Māori-language classifier (Sect. 4.4), we did not want @*user* mentions to be treated as normal text, because usernames do not inherently have a language, and thus should not influence the percentage of Māori text detected in a tweet. The classifier simply disregards all occurrences of “<user>”. In a similar manner, all hyperlinks, beginning with “http://”, were standardised with the text “<link>” to denote their position in the tweet. The final corpus contains 29,826 “<link>” references, with 35.74% of all tweets containing one or more links. Links tend to occur at the end of tweets, whereas user mentions tend to occur at the beginning (mainly because replies are automatically prepended with the username/s of the recipient/s). Note that we did not modify hashtags (#topic) as these have meaningful linguistic content and may be written in te reo Māori (examples include *#tereo* and *#tekupu*; see Sect. 5.4).

#### Removing specific users

When inspecting the number of tweets per user, it was clear that three religious accounts were dominating the corpus. The purpose of their tweets was to disseminate existing translations of the Bible; however, they appear to have been controlled by Twitter bots rather than human moderators. All three accounts were suspended by Twitter and have not been active since 2012/2013. In any case, the content of their tweets does not reflect natural, everyday usage of te reo Māori. As such, we removed all tweets posted by these accounts from the RMT Corpus. Similarly, tweets were removed from a fourth account that explicitly mentioned the word “bot”. These changes reduced the corpus by 60,011 tweets—an astonishing number considering this amounts to 76% of the final corpus.

### Step three: filtering out non-Māori tweets

The third—perhaps most crucial—step was to address the overwhelming presence of non-Māori tweets in our dataset. As noted earlier, most Māori-language tweeters typically only tweet in Māori a fraction of the time, and the majority appear to tweet predominantly in English. There were also many tweets containing both Māori and English text, which is a natural product of bilingual language use, called code-switching. However, because our goal was to create a high-quality corpus of te reo Māori text, rather than to capture instances of code-switching, we needed a way to filter out these tweets, along with those containing no Māori whatsoever. Our process of selecting tweets is much stricter than the *Indigenous Tweets* website; we filter out roughly three-quarters of the tweets that it classifies as Māori.

Our solution for removing non-Māori tweets in the corpus was to calculate the amount of Māori text in each tweet, and then isolate tweets that fell below a certain threshold, allowing for a small margin of error. In order to do this, it was first necessary to identify and extract the Māori tokens (words) used in each tweet. Existing code developed by Te Hiku Media was adapted for this task. We are grateful to Te Hiku Media for allowing us to use code in the *nga-kupu* repository,^21^ in accordance with the Kaitiakitanga Licence.^22^

The *nga-kupu* repository consists of three main Python scripts: *kupu_tūtira, hihira_raupapa* and *auaha_tūtira_tū* (see the repository README file for a description of each). The first of these, *kupu_tūtira*, was deemed to be the most relevant for our purposes. *kupu_tūtira* takes as input some corpus text and returns a list of words that are orthographically consistent with Māori. Specifically, a word is considered to be Māori if it meets the following criteria:

1. The word only contains characters that are part of the Māori alphabet.^23^
2. The word follows consonant/vowel alternation: most Māori syllables take the form (C)(V)V; a few are (C)V1V1V2.^24^
3. The word does not contain any double consonants, excluding ‘ng’ and ‘wh’, which are single consonants in Māori.
4. The word ends in a vowel.

While the *kupu_tūtira* script is effective at identifying Māori words, it does not preclude any non-Māori words that happen to satisfy the above criteria. To resolve this issue, additional items were appended to the stop list, so that they would not be treated as Māori words. These words were determined by manually inspecting the script’s output. We coded anything that was obviously English, including a mixture of formal and informal terms: “autotune”, “amirite” (am I right?), “epitome”, “imitate”, “meringue”, “nope”, “where” and “whiteware” (among others). Our approach relies heavily on having identified Māori-language tweeters in advance (as per step one); otherwise, the algorithm would admit false positives from users who tweet in languages with similar syllable structures.

**Fig. 7:**
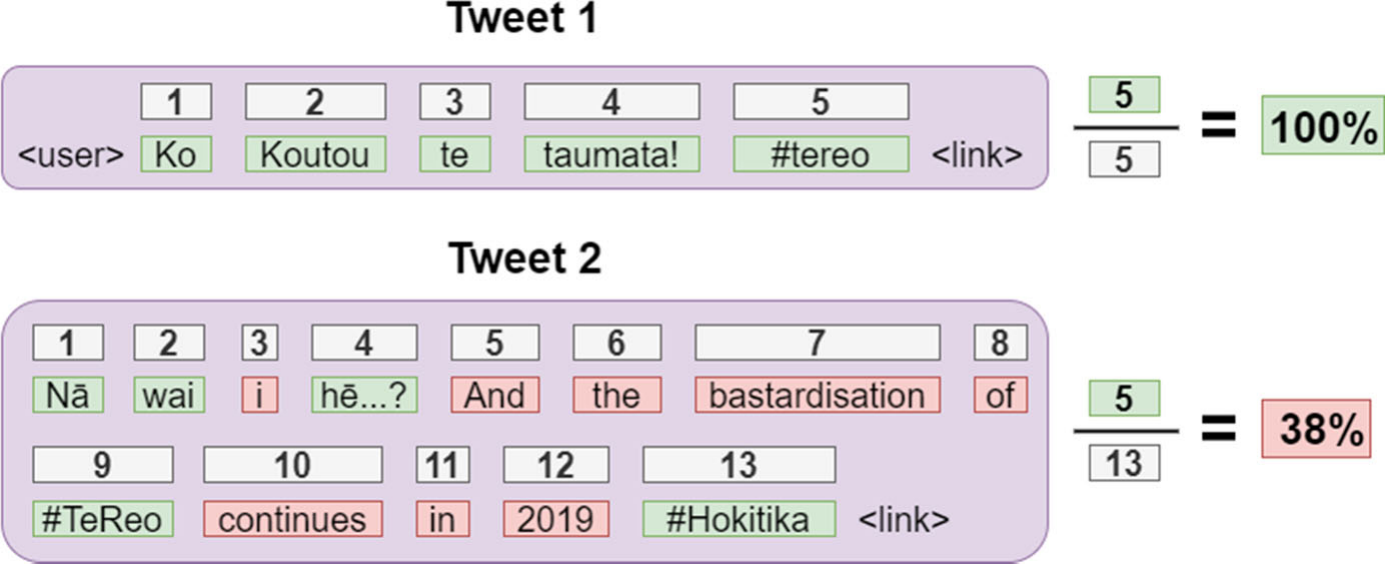
Visualisation showing how the percentage of Māori text was calculated for two different tweets. The grey numbered boxes represent the individual words (tokens) that contribute to the final calculation. Green words are considered to be Māori, whereas red words are considered non-Māori. (Colour figure online)

**Fig. 8:**
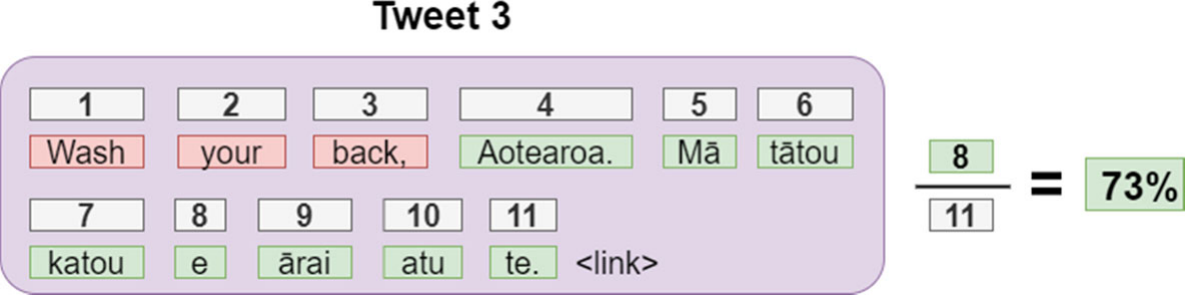
A tweet whose percentage of Māori text only just surpassed the 70% threshold. (Colour figure online)

The *kupu_tūtira* script was then modified to treat each tweet as a separate document/instance. For each tweet, the percentage of Māori text was computed by counting the number of Māori words and dividing this value by the total number of words (see Figs. 7 and 8). All three values (number of Māori words, total number of words and percentage of Māori text) were added to the corpus; this allows analysts to manipulate the data according to the composition of each tweet (e.g. to derive a sub-corpus of tweets with a higher threshold, or to analyse tweets of a specific length).

In order to maximise the amount of data available without compromising its quality, we experimented with a range of different thresholds for the percentage of Māori text in each tweet. Following a holistic examination, we opted for two different thresholds based on the length of the tweet: a minimum of 80% Māori text for tweets containing fewer than seven words, and 70% for tweets containing seven words or more (see Eq. 1). Using these values, we found that 9.4 million tweets did not meet the threshold, and were subsequently removed. This left only 83,491 tweets in the corpus.

1 $$ threshold = \left\{ {\begin{array}{*{20}l}    {0.8,} & {{\text{if}}\;w < 7}  \\    {0.7,} & {{\text{otherwise}}}  \\   \end{array} } \right. $$

To be included in the corpus, tweets are required to have at least 80% Māori text if they contain fewer than seven words (*w*), and at least 70% Māori text if they contain seven words or more.

We believe these values achieve a sensible trade-off between corpus size and accuracy, with all tweets containing mostly Māori text. A higher threshold, such as 90%, would filter out more tweets containing non-Māori words at the expense of also rejecting a greater number of true Māori tweets. There are valid reasons why a Māori-language tweet might receive a lower score than expected. For instance, a tweet may contain some Māori words that were wrongly classified as non-Māori (see Tweet 2). A tweet might also reference one or more non-Māori entities, better known by their English names, such as a person, place or thing. For example, in the following tweet, “St Pauls” is counted as two non-Māori words: “Te hariru a te Kura o Ōwairaka me te Kāreti o St Pauls mutu ana tā rātou tukinga whiringa whāiti i ngā”.

The *kupu_tūtira* script is well-suited to Twitter data for two main reasons. First, the script allows for variations and misspellings, which are a common feature of social media language. For instance, “Aoteroa” is accepted instead of *Aotearoa*, and “whaaaro” is accepted instead of *whaakaro*. These word forms are not unusual on Twitter; however, they are unlikely to be encountered in more traditional genres. Second, there is flexibility concerning double-vowel orthography as an alternative to macron use (e.g. *oo* instead of *ō*). Tweeters sometimes adopt double vowels as a matter of preference,^25^ because they may believe it is a more traditional orthography, or simply because macrons are harder to type. Thus, the script identifies both *Māori* and *Maaori* as words in te reo.

Figure 7 illustrates how the percentage of Māori text was calculated for two different tweets, one which was included in the corpus (Tweet 1) and one which was filtered out (Tweet 2). For Tweet 1, all five words were correctly classified as Māori, and the tweet was assigned a score of 100%. Notice that the < user > and < link > references are excluded from the calculation, which is why they do not have associated indices. This is the case for all tweets, even if the <user> reference is syntactically important. The classifier does not attempt to segment hashtags, so *#tereo* is counted as a single word, despite being made up of *te* and *reo*.

Tweet 2 is an example of a mixed-language tweet that was filtered out of the corpus. Five of its thirteen words were deemed to be Māori, yielding a score of 38%. Notice that the third word, *i*, is not counted as a Māori word, when it should be. This tweet also demonstrates that Māori place names are classified as te reo (e.g. *Hokitika*). Conversely, English place names are considered non-Māori, even if they are part of an otherwise completely Māori tweet.

Unsurprisingly, because our chosen thresholds allow 20–30% leeway, there are some tweets that contain a small amount of non-Māori text. Tweet 3 (Fig. 8) is an example of a tweet that only just meets the 70% threshold. Composed of three English words and eight Māori words, this tweet features a COVID-19 slogan, “Mā tātou katoa e ārai atu te” (“We can all prevent this”). The first word (“wash”) is used as a humorous play on “watch”. Despite the presence of English, Māori is still the prevailing language in the tweet; an English-only speaker would struggle to understand its meaning. This is true of many other tweets in the corpus that also have relatively low Māori percentages.

While the *kupu_tūtira* script was highly effective at removing both non-Māori tweets and tweets containing large quantities of English, it has minor limitations that need to be considered. Most notably, some English words share the same form (spelling) as Māori words, meaning that they are ambiguous cases. Ideally, the classifier would maintain awareness of the surrounding context of these words to decide whether they are Māori (e.g. “hope” is likely to be English if surrounded by English words, but Māori if surrounded by Māori words). However, the classifier treats *all* instances of a word as being either Māori or non-Māori, regardless of actual usage in a particular context.

If a Māori word contains more than three letters and shares the same form as an English word, it is always considered to be Māori. Examples of such words are *hop*e, *take*, *more* and *mate*. Although these words tended to be used much more frequently in English, especially because the input dataset contained more English text than Māori, we deduced that these words were more likely to be the difference between a valid Māori tweet being rejected from the corpus than an English tweet being wrongly included.^26^

Conversely, Māori words containing fewer than three letters and sharing the same form as English words were mostly assumed to be non-Māori.^27^ This includes the words *i*, *a*, *to* and *no*, which are all very common in English, but which are also common function words in Māori (see Tweet 2). We could have modified the *kupu_tūtira* script so that these were always treated as Māori, but this would have resulted in more English tweets being added to the corpus. This reinforces the need for allowing some flexibility in the percentage of Māori text, because a Māori tweet will invariably be penalised if it contains many instances of these words.

In addition, the *kupu_tūtira* script cannot always distinguish between legitimate Māori words (or their misspellings) and completely made-up words (e.g. *tatata* vs “tatatata”). Fortunately, these made-up words tend to be highly infrequent, which means their impact is negligible. We could have verified the legitimacy of these words using Te Hiku Media’s *hihira_raupapa* script, which compares them with the online Māori dictionary,^28^ but we did not want to forego the flexibility that came with permitting common variations and misspellings encountered on Twitter.

#### English versus Māori-language tweeting

Less than 1% of the 9.5 million tweets that were used as input for step three had sufficient Māori text to be included in the corpus. The overwhelming majority of discarded tweets were written in English, showing that the English language dominates the Twittersphere even among the cohort of reo Māori tweeters. Qualitative research would be needed to understand the reasons why Māori-language users choose to tweet in Māori or English in a particular context. For example, an individual’s decision to tweet in Māori (or not) might depend on an awareness of their audience, their own proficiency, ideology, the topic of conversation, or a desire to conform with societal norms and expectations. Interviews could be conducted with Māori-language tweeters to gain deeper insight into the rewards and challenges of tweeting in te reo, and how these weigh up against tweeting in English.

### Step four: removing formulaic tweets

While significant progress had been made in filtering out unwanted noise, manual inspection of the corpus revealed that some tweets which had been classified as Māori still did not reflect natural usage of te reo. We performed a fourth and final step, removing so-called ‘formulaic’ tweets, to address this.

When sorting the data alphabetically, we noticed a considerable number of tweets in the corpus whose content was very similar, or even identical. However, this was to be expected, due to the presence of short tweets containing generic phrases and expressions (e.g. “Kia ora e hoa”, “Rā whānau ki a koe”, “ka mau te wehi”), which are frequently used by both the same and different users. It makes sense to keep these tweets in the corpus, because they reflect authentic language use and may provide insights into the frequency of various syntactic patterns and recurrent constructional schemas.

However, contrary to this, we also encountered a less desirable type of similar tweet, which clearly did *not* reflect natural speech. The content in these tweets appeared to have been created according to some sort of template. Typically, these tweets were identical, except for minor differences in formatting, such as capitalisation, punctuation, numbers, users and links. For example, the tweets “Te Karere Pānui—Rāpare 13 Hakihea 2012—<link>” and “Te Karere Pānui—Rāpare 06 Hakihea 2012—< link>” are identical apart from the numbers after *Rāpare* (13 and 06). Because these tweets can occur a large number of times, their inclusion in the corpus would unfairly inflate associated counts.

To resolve this issue, we transformed tweets into a ‘comparison string’, and kept only one instance of the (original) text for any longer strings that matched. Each comparison string was generated by converting all letters in the tweet to lower-case, and stripping any punctuation, numbers and <user> or <link> references. The resulting strings were then compared with all other tweets *by the same user*. We observed that the (undesirable) synthetic tweets tended to be longer than the (desirable) common phrases, which seemed to mostly contain four to six words. As such, we decided to keep all instances of similar tweets containing fewer than seven words, and only the first instance of any similar tweets by the same user containing seven words or more. This also served to remove accidental repostings of longer tweets (e.g. two identical tweets posted within a minute of each other). 3,926 tweets were removed accordingly.

We removed a further 547 formulaic tweets that were not detected during this process, because they only occurred once. This mostly included announcements for events, programs and competitions, such as kapa haka results (e.g. “Kākahu: 3rd Te Rōpū Manaaki, 2nd Te Rōpū Kapa Haka o Whaitara, 1st Te Wharekura o Hoani Waititi #hakawhangarei”). Such tweets contained almost no Māori text other than proper nouns. Tweets were also removed from an account that was dedicated to posting a “Māori word of the day” (e.g. “tapahi(a), tapatapahi(a): tapahi(a), tapatapahi(a): cut, dice. E tapatapahia ana ngā aniana e ia. The… < link > #tekupu”). The content in these tweets is not unique to Twitter, being directly copied from (and linked to) another site, much like the Bible translations in Sect. 4.3.1. After removing a total of 4473 tweets in step four, 79,018 tweets remained, which we believe (for the most part) contain original and authentic Māori text.

## Preliminary analysis of the RMT Corpus

In this section, we present a preliminary analysis of the RMT Corpus. We start by providing an overview of the corpus, then analyse the most popular words, topics and hashtags, and finally report the number of tweets and users per year, with particular focus on the ten most prolific tweeters. We intend to build on this analysis by creating visualisations for exploring the RMT Corpus in future work.

A basic overview of the RMT Corpus is given in Table 2. The corpus encompasses nearly 80,000 tweets, comprising one million words, written by 2,300 users. Note that hyphenated words are counted as a single token (e.g. each instance of *here-turi-kōkā* counts as one word). “<link>” and “<user>” references are excluded from the total word count, whereas numbers and hashtags are not.

**Table 2:** Key summary statistics for the RMT Corpus

Description	Value
Number of Tweets	79,018
Number of Tokens (Words)	1,007,652
Number of Users (Tweeters)	2302
Average Tweet Length^a^	12.75 words
Average Tweets per User	34.33
First (Oldest) Tweet	25 May, 2007
Last (Most Recent) Tweet	11 December, 2020
Time Period	~ 13.5 years

^a^Note that Twitter’s character limit increased from 140 to 280 characters after November 2017

### Top 100 words by frequency

**Fig. 9:**
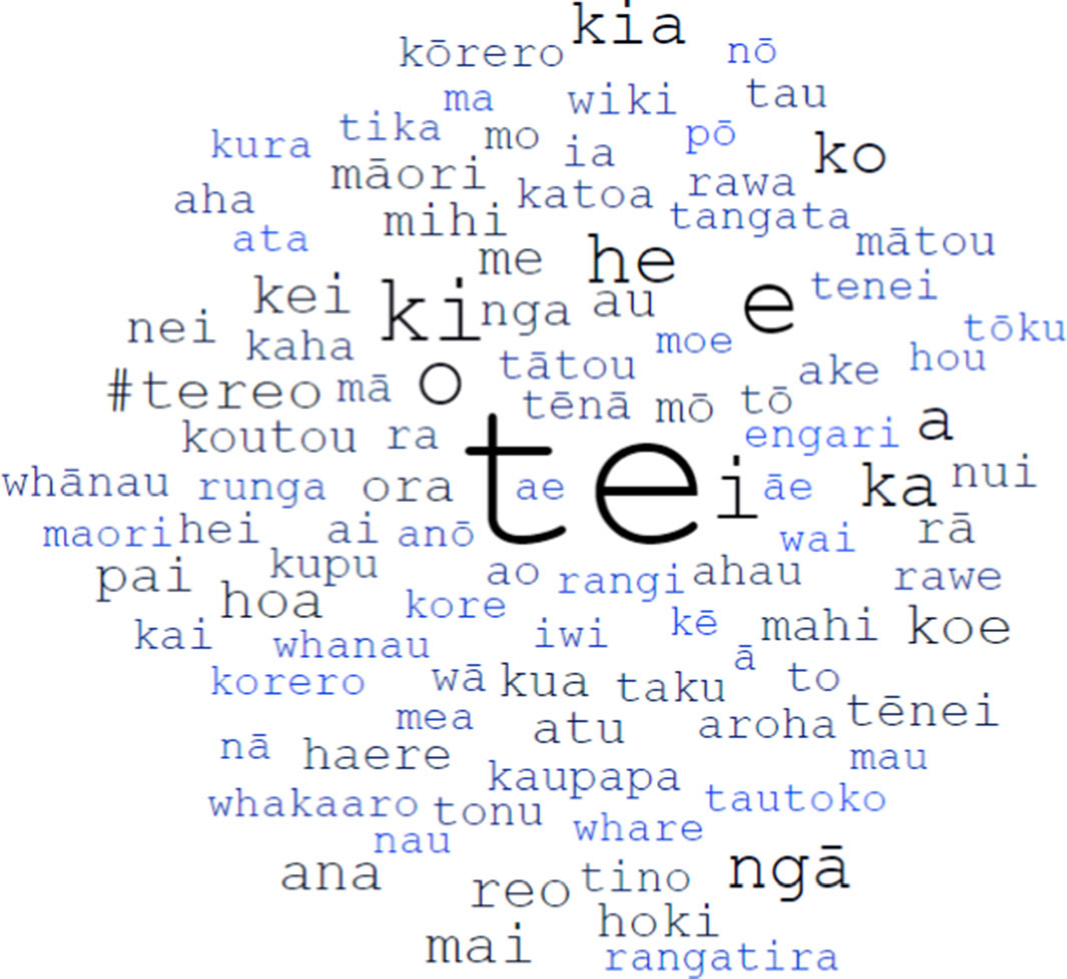
Word cloud showing the top 100 words in the RMT Corpus, where size and hue are proportional to frequency. Drawn using https://worditout.com

We now examine high frequency vocabulary in the RMT Corpus. Figure 9 is a word cloud showing the 100 most common tokens (words) in the corpus. The size and darkness (hue) of each word are proportional to its overall frequency. When performing these calculations, all tokens were converted to lower-case but otherwise left unchanged. As a result, macron and non-macron variants of the same word (e.g. *māori* and *maori*) were not consolidated, but rather treated as separate entities. An exhaustive word list, with frequencies for all tokens in the RMT Corpus (not just the top 100), is available on the companion website.^29^

Looking at Fig. 9, it is clear that *te* is by far the most prolific word in the corpus. This is not surprising, given that *te* in Māori has a similar role to the definite article (“the”) in English (albeit confined to singular noun phrases): it serves a ubiquitous grammatical function that does not add any meaning to a sentence or clause (see Harlow, 2001, p. 309). There is only one hashtag that appears in the top 100 tokens, namely *#tereo*, which is ranked 13th overall, with 10,486 occurrences. 21 words in the top 100 contain one or more macrons, most notably: *ngā* (‘the’, plural; 7th; 15,294 instances), *māori* (‘of Indigenous language/people’; 27th; 5,848 instances), *rā* (‘day/sun’/various meanings; 28th; 5,840 instances) and *tēnei* (‘this’, by speaker; 30th; 5,576 instances).

### Top ten words by frequency

**Fig. 10:**
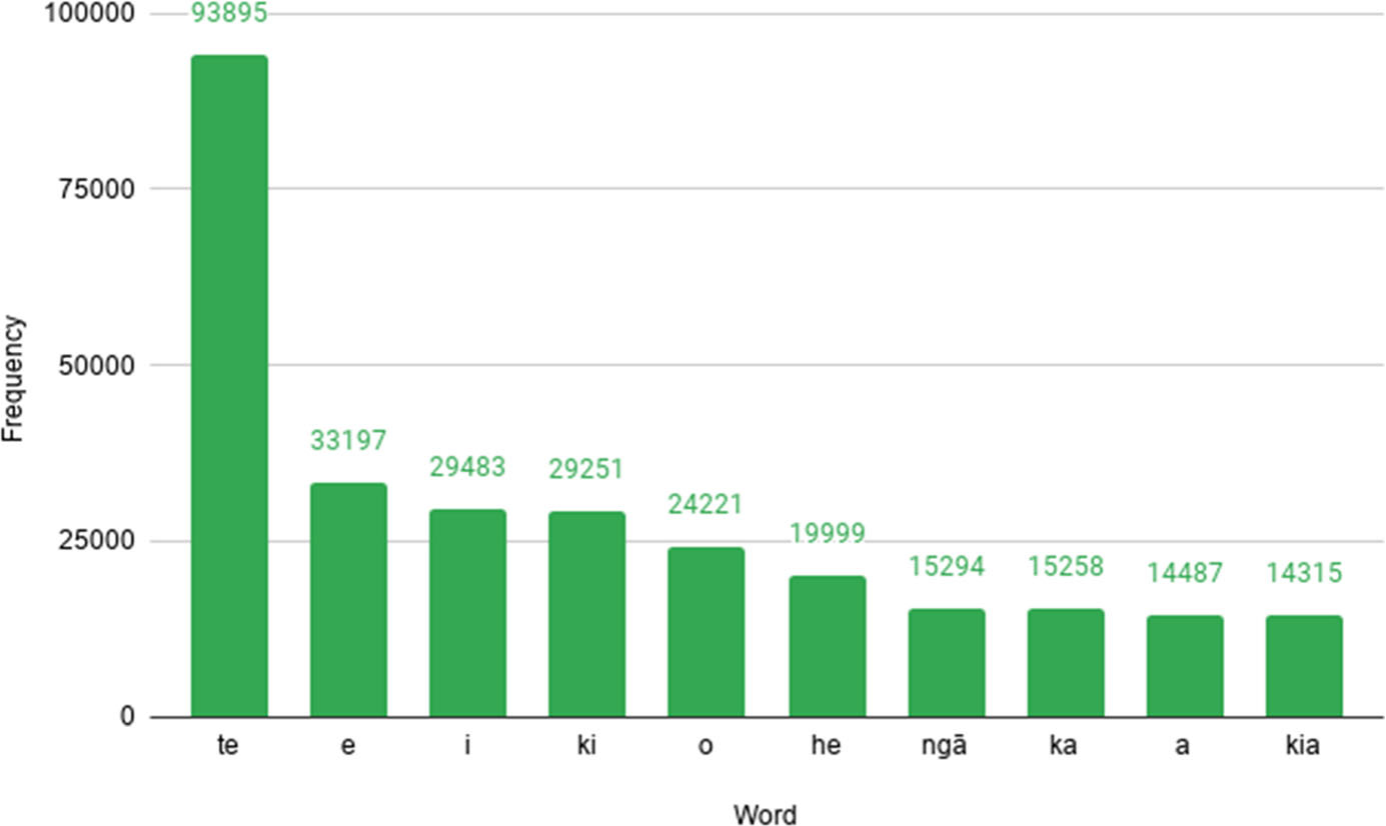
The ten most frequent words in the RMT Corpus, which are all function words, and their associated frequencies of occurrence. The most common meanings of these words are: *te* ‘the’ (singular), *e* (various meanings), *i* (various meanings), *ki* ‘to/at’, *o* ‘of’, *he* ‘a/some’, *ngā* ‘the’ (plural), *ka* (verbal particle), *a* (nominal particle), and *kia* (various meanings)

Figure 10 drills down into the ten most frequent tokens in the RMT Corpus. As with English,^30^ these are all function words (particles) rather than content words. Their role tends to be as determiners, or as words signalling tense, aspect and mood. Like all function words in Māori, they are very short, containing no more than two morae. It is worth noting that the frequency of *i*, *he* and *a* could be conflated with English (arising from tweets with a small number of non-Māori words), although one would still expect these instances to be predominantly Māori.

An interesting hypothesis is that character limits imposed by Twitter might influence the frequency with which these words are used. Boot et al. (2019) found that Dutch-language tweeters adapted the content of their tweets to overcome character limit constraints, changing sentence structure and word forms as necessary. Twitter’s character limit increased from 140 to 280 characters in November 2017, so tweets before and after this period may have noticeable linguistic variation (as was investigated in the aforementioned study). Further analysis would be needed to determine whether Māori function words are used differently (e.g. more sparingly) on Twitter, compared to other genres.

### Top ten content words by frequency

**Fig. 11:**
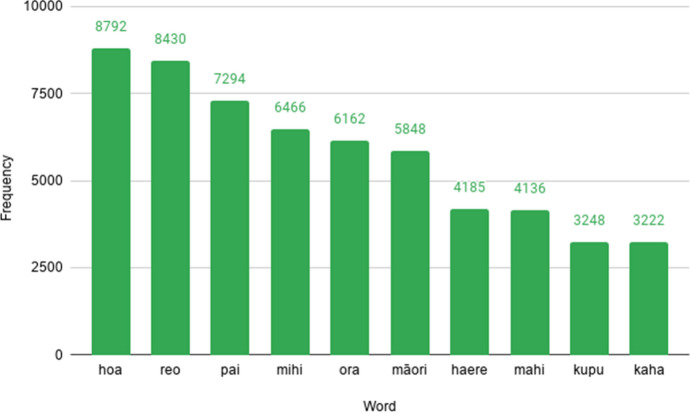
The ten most frequent content words in the RMT Corpus and their associated frequencies of occurrence. Common meanings of these words are: *hoa* ‘friend’, *reo* ‘language’, *pai* ‘good’, *mihi* ‘to greet’, *ora* ‘healthy’, *māori* ‘Indigenous’, *haere* ‘come’, *mahi* ‘work’, *kupu* ‘word’ and *kaha* ‘strong’

As the ten most frequent words in the corpus are all function words, we now provide an overview of the top ten content words (Fig. 11). Many of these words appear to be used in tweets whose discourse function is to increase solidarity, as they are typically used in greetings (e.g. *hoa* ‘friend’, *pai* ‘good’, *mihi* ‘to greet’, *ora* ‘healthy’, *kaha* ‘strong’). Other content words relate more directly to aspects of Māori culture or language (e.g. *māori* ‘indigenous’, *reo* ‘language’ and *kupu* ‘word’). We excluded the hashtag *#tereo* as this is considered in the next section.

### Hashtags in the RMT Corpus

Hashtags are a pervasive feature of Twitter and are widely used in search engines. Users often include hashtags in their tweets to contribute to a wider discussion, introduce new topics, extend conversations or emphasise key points in their tweets. Although introduced to Twitter in August 2007, the first hashtag does not appear in the RMT Corpus until 2009. In total, there are 46,195 hashtag ‘tokens’ in the corpus, including 10,441 distinct types, although only 3073 of these appear more than once.^31^ These hashtags are written in both Māori and English, and tell us about the general interests and motivations of Māori tweeters.

**Fig. 12:**
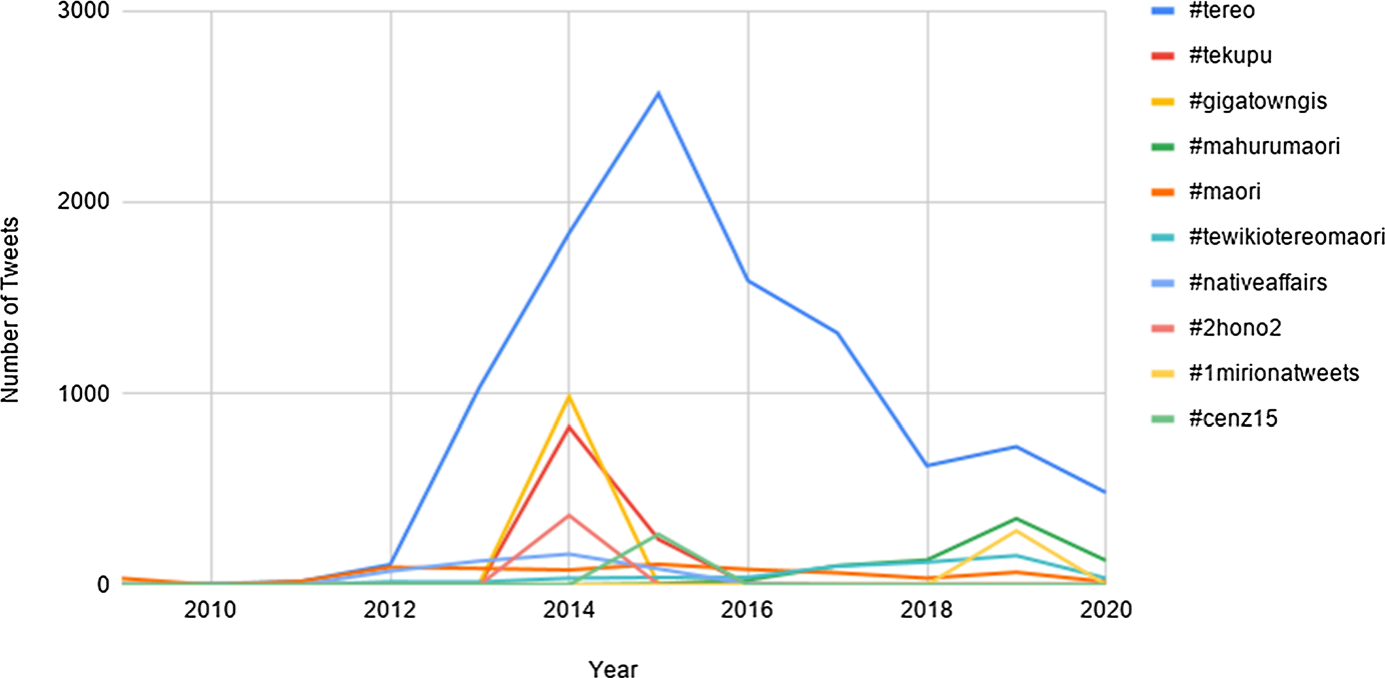
Diachronic trajectory of the ten most common hashtags in the RMT Corpus. (Color figure online)

Figure 12 shows the diachronic trajectory of the ten most frequently used hashtags in the RMT Corpus. Semantically, the hashtags pertain mostly to topics concerning Māori language and culture (e.g. *#tewikiotereomaori* refers to Māori Language Week), which was also reported as a prominent theme in relation to hybrid hashtags (Trye et al., 2020). Note that the graph does not include instances of hashtags whose timestamp is unknown (roughly 3% of the data). There are likely to be other tweets (both Māori and non-Māori) that also use these hashtags, but which are not included in the corpus. The data for 2020 is also less reliable than in previous years, for reasons outlined in Sect. 4.2. Consequently, the patterns shown in Fig. 12 do not necessarily characterise the overall distribution of each hashtag on Twitter. Nevertheless, they provide an indication of when the ten hashtags were most frequently being used, especially with Māori content, and whether they persisted across multiple years. Only half of the top ten hashtags are recorded at least once in the corpus in 2020 (namely, *#tereo*, *#mahurumaori*, *#maori*, *#tewikiotereomaori* and *#1mirionatweets*).

The most dominant hashtag in the corpus is *#tereo* (dark blue), which had more uses than any other hashtag between 2010 and 2020. In 2015 alone, this hashtag appeared in 2,569 tweets. In fact, for any given year between 2014 and 2017 (inclusive), *#tereo* is used more often than the total frequency (i.e. all years combined) for each of the other nine hashtags.

The hashtags *#gigatowngis*, *#tekupu* and *#2hono2* all peaked in 2014. *#gigatowngis* refers to a national competition to receive city-wide funding for ultra-fast broadband, enabling the winner to become New Zealand’s first “Gigatown”.^32^*#2hono2* is used in relation to a fortnightly Twitter chat, facilitated by Connected Educator New Zealand (CENZ), with a view to “connecting Māori medium learning environments, whānau, educators, & communities throughout Aotearoa”.^33^ This hashtag appears in 364 tweets in 2014, and then only twice in 2015. *#cenz15* refers to the 2015 iteration of the CENZ programme, and, as the name suggests, is used exclusively in 2015.

The hashtags *#mahurumaori* and *#1mirionatweets* both peaked in 2019 (*mahuru* means ‘September’ and *miriona* means ‘million’). This is not surprising, because they were two of the three official hashtags for a nation-wide campaign launched in September 2019, which aimed to achieve one million te reo Māori tweets. Following the launch, Te Māngai Pāho (the Māori Broadcast Funding Agency) released topics every day during the month of September, which New Zealanders could then weave into their content. It appears that the milestone of one million te reo tweets is still a long way off, with the hashtags *#mahurumaori* and *#1mirionatweets* collectively appearing only 1,079 times in the corpus. The third hashtag for the campaign, *#1mirionatihau*, was ranked 15th overall, with 208 occurrences.

Table 3 provides additional statistics about the top ten hashtags, including their raw frequency (in the corpus) and the number of tweeters who used them at least once. An example of each hashtag is also given to provide additional context about how it is used. *#tereo* is not only used in the most tweets, but also by the most distinct users (358). *#tewikiotereomaori* is ranked sixth overall, but is used by the second highest number of users (243). The hashtag *#cenz15* is only employed by eight different tweeters, but is used more frequently among them, with an average of 33 instances per tweeter.

**Table 3:** Information about the ten most frequent hashtags in the RMT Corpus, including their frequencies and an example of each

Rank	Hashtag	Raw frequency	Distinct users	Example
1	*#tereo*	10,512	358	Ka tangi te pīpīwharauroa, ko te karere a Mahuru #pēpeha **#tereo** < link >
2	*#tekupu*	1168	104	Kia horapa ēnei pānui ēnei ki te rohe o Whanganui **#tekupu** < link >
3	*#gigatowngis*	1027	47	Tera, ka huri ki te ara etahi, ko Titirangi i kora **#gigatowngis** < link >
4	*#mahurumaori*	790	127	#22pressupchallenge, **#mahurumaori**, ra waru ka tukuna hei arohanui ki ku matua kk ka riro atu ki te p. < link >
5	*#maori*	718	168	Tirohia ngēnei! Te wai korarī nō te rohe o Maniapoto #inu **#Māori** < link >
6	*#tewikiotereomaori*	597	243	Kia Pai Tō Haere. He rauemi marautanga. **#tewikiotereomāori** < link > < link >
7	*#nativeaffairs*	501	91	**#NativeAffairs** ka aroha ki aua kura
8	*#2hono2*	374	29	Whoop whoop! Whakarauora te reo māori, kia eke ki tōna pane-kiretanga **#2hono2**
9	*#1mirionatweets*	289	53	Kia kaha tātau ki te kōrero i te reo Māori, ahakoa ki whea, ahakoa ko wai! **#1MirionaTweets** < link >
10	*#cenz15*	266	8	Kei runga noa atu koe e hoa! #tereo **#CENZ15** < link >

### Basic user statistics

**Fig. 13:**
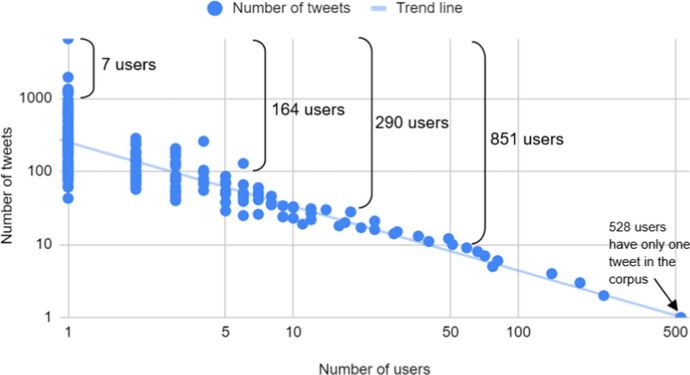
The number of tweets per user in the RMT Corpus, shown on a logarithmic scale and revealing a highly skewed distribution

Different users have different numbers of tweets in the RMT Corpus, depending on how often they tweet in te reo. A diachronic analysis of the ten most active tweeters is given in Sect. 5.7, but we first examine the overall composition of the corpus at a macro-user level. Figure 13 shows that the distribution of tweets by Māori-language users is highly skewed, with a small proportion of users contributing many tweets to the corpus, and a large proportion having only one or a few. Thus, a small number of Twitter users account for the majority of Māori-language tweets, which also matches the distribution on the *Indigenous Tweets* website. Specifically, the top eight users account for a fifth of the data in the RMT Corpus; the top 24 users account for a third; and the top 70 account for half.

**Fig. 14:**
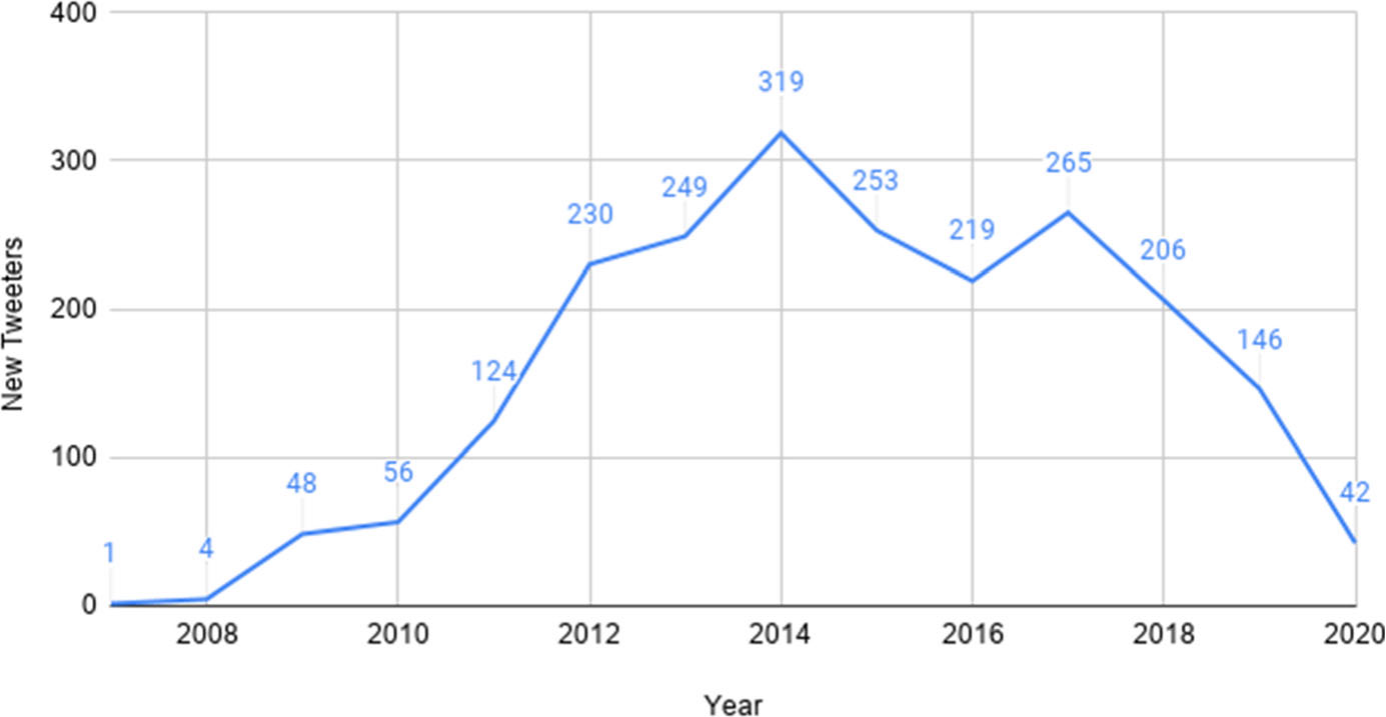
The number of *new* users per year in the RMT Corpus, representing growth in the Māori-language Twitter community over time

Figure 14 plots the number of new Māori-language tweeters per year in the RMT Corpus. In this graph, tweeters are only counted the year that their very first tweet appears in the corpus. This provides an indication of the extent to which the community of Māori-language tweeters has grown over time, although there is no guarantee that tweeters from past years are still active, and some users may have deactivated previous accounts and created new ones, thereby distorting the figures. Overall, we can see that there is some degree of positive growth across all years (i.e. there is at least one new Māori-language tweeter per year), but that the period from 2012 to 2018 experienced the most growth, with more than 200 new users per year. 2012 had the largest surge compared to the previous year. The uptake of new users in later years has been comparatively slow, with only 42 new users recorded for 2020 (also representing the largest *decrease* with respect to the previous year; however, this could also be an artefact of the *Indigenous Tweets* classifier not having been updated recently). There are 140 tweeters for whom no tweet timestamps are available, whose data is therefore not shown.

### Diachronic analysis of tweets per year

**Fig. 15:**
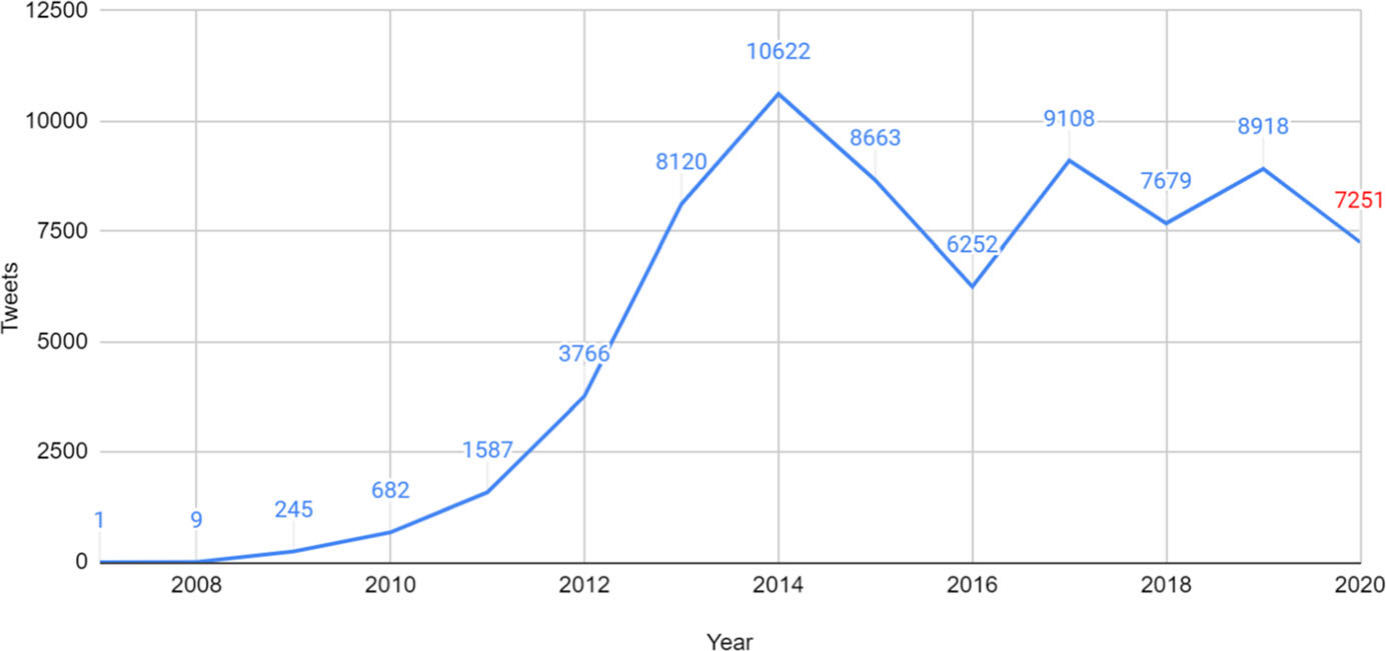
The number of tweets per year in the RMT Corpus

**Fig. 16:**
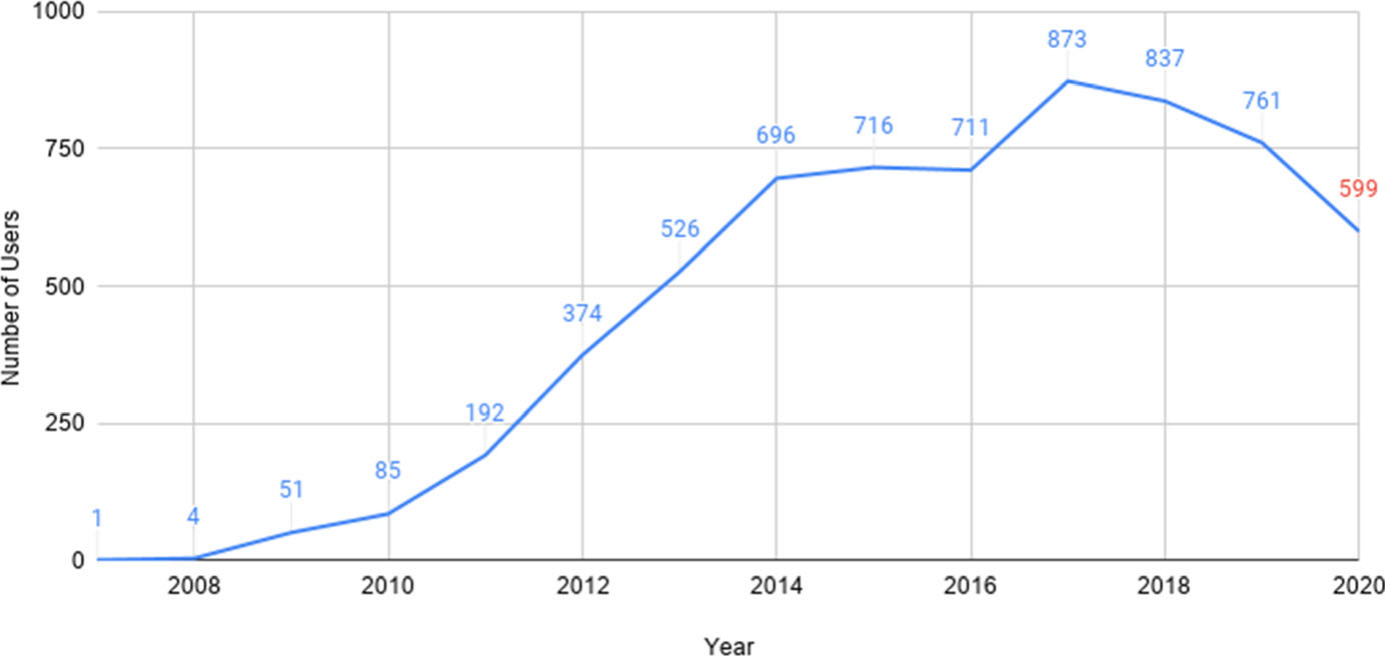
The number of active users per year in the RMT Corpus

Next, we discuss how the number of Māori-language tweets and active users in the RMT Corpus changes over time, and comment on related patterns and trends. Figure 15 shows the distribution of tweets per year, whereas Fig. 16 shows the active users. The number of tweets and active users appear to be positively correlated (i.e. as the number of users increases, so does the number of tweets in the corpus).

It is immediately apparent from Fig. 15 that very few Māori tweets included in the corpus were posted during the first few years of Twitter (to be precise: none in 2006, one in 2007, 9 in 2008 and 245 in 2009). The volume of Māori tweets increases very steeply over the next five years, reaching its highest point, 10,622 tweets, in 2014. Following this, the number of tweets declines for a couple of years, and then remains relatively stable, with reasonably small fluctuations from year to year. By 2020, although the number of tweets is roughly only two-thirds of what it was in 2014, there are still significantly more tweets than there were pre-2013, a trend which looks likely to continue.

It should be noted that 6,115 tweets (7.74% of the corpus) are omitted from Fig. 15 because we were not able to retrospectively retrieve their timestamps. Therefore, a limitation of these findings is that they may not reflect actual trends for the entire corpus, especially if a large portion of excluded tweets were posted in the same year. In addition, a limitation of the data for 2020 is that, due to changes in how the tweets were collected, we could not gather tweets from some users whose data was previously available. This means that the figure reported for 2020 is likely to be an underestimate of the actual amount, and we must therefore proceed with caution.

Figure 16 shows that, even though the number of new users in 2020 is relatively low (Fig. 14), there is still a large number of active tweeters (599, which is a conservative measure, given the less reliable data for 2020). Comparing Figs. 15 and 16, the number of tweets and active users both increase rapidly between 2009 and 2014. However, unlike the number of tweets, the number of active users in 2016 only decreases very slightly. Thus, it would appear that, in 2016, roughly as many users were tweeting in Māori (compared to 2015), but they were posting fewer tweets each. The number of active users declines steadily over the last four years, from 873 users in 2017 to 599 in 2020.

### Diachronic analysis of the ten most active tweeters

**Fig. 17:**
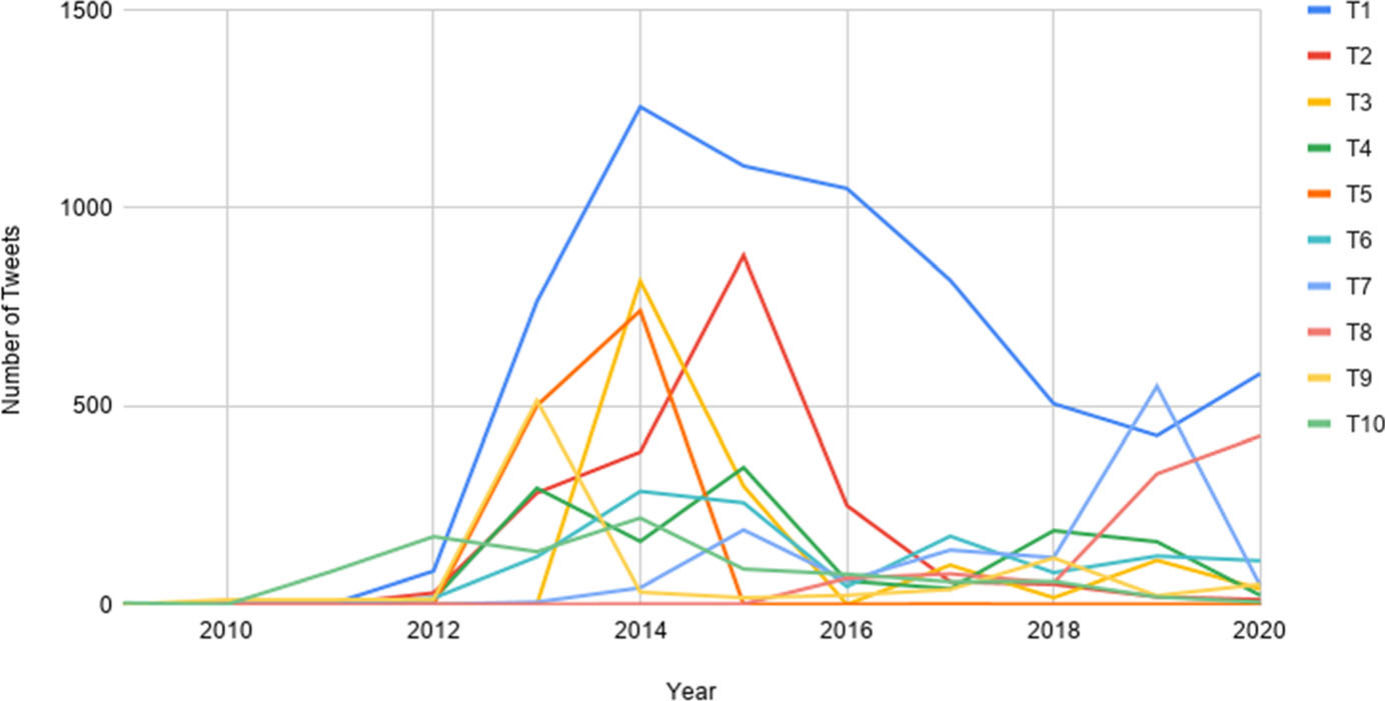
Diachronic trajectory of the ten most frequent tweeters in the RMT Corpus. (Color figure online)

Having considered the overall number of tweets and users per year, we now analyse diachronic trends of the ten most prolific tweeters in the RMT Corpus (Fig. 17). One of the most striking observations is that many of these users have not been as active in recent years. Most of the ten most prolific tweeters’ Māori-language activity spiked between 2013 and 2015, after which their volume of tweets steadily declined. While the reasons for this are not clear, it may be because the individuals concerned are now using Twitter less often (perhaps favouring other social media platforms), and as such, posting fewer tweets. However, it is worth noting that most of these users continue to tweet in te reo Māori (in 2020), albeit to a lesser extent. The prevalence of tweets between 2013 and 2015 matches and, indeed, goes some way towards explaining, the overall temporal trends seen in Fig. 15.

## Downloading the RMT corpus

The RMT Corpus can be downloaded for academic use. Please follow the instructions, given in both Māori and English, on our companion website: https://kiwiwords.cms.waikato.ac.nz/rmt_corpus/. In order to comply with Twitter’s terms of service, we have only released the IDs and selected metadata for each tweet. Some tweets in the RMT Corpus are no longer publicly available and, as such, cannot be downloaded from Twitter. The statistics and analyses presented in this paper reflect version one of the corpus (rmt-corpus-v1.csv); however, we intend to supplement our data with new, more recent tweets in the future. See the project GitHub repository for further details: https://github.com/Waikato/kiwiwords/tree/master/rmt_corpus.

## Conclusions and future work

The main contribution of this work is a publicly available corpus of reo Māori tweets, which constitutes the largest known collection of Māori-language data from social media. The RMT Corpus was compiled by gathering tweets from users identified by the *Indigenous Tweets* classifier. Although 11 million tweets were initially collected, only 79,018 of these (0.72%) satisfied our stringent criteria for inclusion in the corpus. Tweets of many different types, including non-Māori tweets, short tweets, retweets, automated tweets and formulaic tweets, were all filtered out at various stages, in order to improve corpus reliability. The final corpus comprises roughly one million words, written by 2302 users.

Our preliminary analysis examined high-frequency words and hashtags in the RMT Corpus. Initial findings suggest that Māori-language tweets tend to reference topics relating to Māori language and culture. These tweets are often used to increase solidarity and signal community affiliation. We also investigated the number of tweets and active users per year, paying special attention to the ten most prolific tweeters, who account for more than a fifth of the data in the corpus.

The RMT Corpus opens exciting avenues for future work. It demonstrates how te reo Māori is used in authentic, communicative contexts, which makes it valuable for linguistic analysis and the creation of teaching materials. The corpus constitutes a rich, multi-dimensional dataset, whose user metadata could be used to inform decisions about language revitalisation initiatives, especially in digital contexts. From a linguistics perspective, the RMT Corpus can be used (among other things) to study high frequency words, collocations and constructional schemas. From a sociolinguistic viewpoint, it would be interesting to investigate whether the corpus captures any recent changes in the Māori language, such as a shift in the use of pronouns and possessive markers (A/O categories) or voicing distinctions. Given that Māori is experiencing a wave of changes, whereby L1 *kaumātua* (elders) are using different constructions from L2 young Māori-language learners, it would be interesting to investigate how these changes might be evolving in a platform which largely appeals to a younger audience. As a unique source of social media data, the RMT Corpus could be fruitfully compared with other, more traditional genres of Māori text (including those detailed in Sect. 3.1), for instance using a keyword analysis (following Hardie, 2014). Analysts could also study inter-speaker variation within the corpus, both synchronically and diachronically, by drawing on the rich array of tweet and user metadata (although care would need to be taken to derive a balanced subset for analysis). Qualitative research is needed to determine the reasons why Māori-language speakers choose to tweet in Māori (or not) in a particular context, and to shed light on the rewards and challenges of doing so. Furthermore, we anticipate that the RMT Corpus will facilitate the development of new NLP resources for the Māori language, including text-to-speech, speech-to-text and auto-completion technologies.

As has been emphasised throughout the paper, the RMT Corpus was designed to contain almost exclusively Māori text. An empirical evaluation of our language identification scheme is left for future work. Our next step will be to create a corpus of Māori/English code-switching from the discarded tweets (among others), by targeting those with mixed-language text. We believe this will have useful applications in NLP, such as helping to improve New Zealand English text-to-speech, by capturing how Māori is spoken in everyday contexts (i.e. often interspersed with English).

Finally, the RMT Corpus could benefit from extra data, perhaps even collecting this in real-time. Tweets posted after December 2020 would make a welcome addition to the corpus, as would Māori-language data from other social media platforms, such as Facebook and Instagram. We currently discard bilingual tweets, even if they contain Māori text that could stand alone, so it might be beneficial to incorporate this data in the future. Having as much high-quality language data as possible is vital to ensure the accuracy and subsequent proliferation of te reo Māori in NLP applications and environments.

## Supplementary Information

Below is the link to the electronic supplementary material.

Supplementary file1 (DOCX 19 kb)

## Data Availability

Instructions for downloading the RMT Corpus are available at the companion website: https://kiwiwords.cms.waikato.ac.nz/rmt_corpus/.

## Footnotes

1 In this paper, the terms *Māori*, *te reo* and *te reo Māori* are used interchangeably. We use macrons to denote long vowel sounds, following Te Taura Whiri’s (2012) *Guidelines for Māori Language Orthography.*

2 The 2018 New Zealand census reports a total of 185,955 Māori speakers: https://www.stats.govt.nz/information-releases/2018-census-totals-by-topic-national-highlights-updated.

3 See https://www.teaomaori.news/1mil-tweets-te-reo.

4 This is an annual government-sponsored initiative, now officially called *Te Wiki o te Reo Māori*.

5 See https://translate.google.com/.

6 See http://community.nzdl.org/macron-restoration/jsp/mi/main.jsp.

7 See https://papareo.nz/, https://kaituhi.nz/ and https://koreromaori.io/.

8 See https://papareo.nz/#kaitiakitanga.

9 We expect researchers to adhere to the international data principles of FAIR (Findable, Accessible, Interoperable and Reusable) and CARE (Collective Benefit, Authority, Responsibility and Ethics).

10 See http://techiaith.cymru/data/corpora/twitter/?lang=en.

11 Copied directly from http://techiaith.cymru/data/corpora/twitter/?lang=en.

12 See http://indigenoustweets.com.

13 See https://developer.twitter.com/en/docs/twitter-api.

14 See http://indigenoustweets.blogspot.com/2011/03/welcomefailte.html.

15 The RMT Corpus comprises only a quarter of these tweets, for reasons explained in Sects. 4.3 and 4.4.

16 This is a reasonable assumption to make, given that all adult Māori speakers are bilingual, and English is the dominant language of New Zealand.

17 Twitter suspends accounts that violate its rules: https://help.twitter.com/en/rules-and-policies/twitter-rules.

18 We previously had premium access to the Twitter API, which provided access to historical tweets from protected and deleted accounts up to the beginning of 2020, but we were not able to collect such tweets throughout 2020.

19 See https://developer.twitter.com/en/docs/twitter-api/conversation-id.

20 See https://help.twitter.com/en/managing-your-account/about-twitter-verifiedaccounts#:*:text=Established%20digital%20content%20creators%20may,below%20for%20other%20influential%20individuals.

21 See https://github.com/TeHikuMedia/nga-kupu.

22 *Kaitiakitanga* is a term meaning ‘guardianship’, as opposed to ‘ownership’. See https://github.com/TeHikuMedia/corpora#license-kaitiakitanga.

23 The Māori alphabet is derived from the Roman script and consists of five vowels (*a, e, i, o, u*) and ten consonants (*h, k, m, n, ng, p, r, t, w, wh*). A diacritic mark called a macron (tohutō) is placed over vowels to indicate a lengthened vowel sound.

24 See http://www.maorilanguage.info/mao_vocab_faq.html.

25 This convention was popularised by Bruce Biggs in the 1960s, due to his computer’s inability to type the macron character (personal communication to Te Taka Keegan, 29 September, 1992).

26 An example of a tweet where this is *not* the case is: “Kia ora e hoa, *hope* it’s useful!!”, where *hope* is regarded as a Māori word, and the tweet receives a final score of 5/7 (71%) instead of 4/7 (57%).

27 *He* being a notable exception.

28 See https://maoridictionary.co.nz/.

29 See https://kiwiwords.cms.waikato.ac.nz/rmt_corpus/.

30 See http://www.kilgarriff.co.uk/BNClists/lemma.num.

31 Unlike the content and function word analyses above, the number of hashtag types (in this section) was calculated after converting all letters to lower-case and removing macrons. It made sense to merge these different forms, because searching for the hashtag *#Maori* on Twitter will also return results for *#maori* and *#Māori*: https://twitter.com/search?q=%23Maori&src=typed_query. Moreover, hashtags did not permit macrons in the early days of Twitter.

32 See https://company.chorus.co.nz/gigatown.

33 See http://connectededucator.org.nz/event/twitter-chat-2hono2-te-tuhonohono-i-te-iwi-maori/.

## Code availability

Code for cleaning, analysing and hydrating the corpus is available at the following GitHub repository: https://github.com/Waikato/kiwiwords/tree/master/rmt_corpus.
