# Supplementary material for: Harnessing Indigenous Tweets: The Reo Māori Twitter corpus
Source: Lang Resour Eval. 2022 Feb 14;56(4):1229–68. doi: 10.1007/s10579-022-09580-w (PMC8852919; doi:10.1007/s10579-022-09580-w)
Supplement: Supplementary file 1 — Supplementary file1 (DOCX 19 kb) [file 10579_2022_9580_MOESM1_ESM.docx]

**Harnessing Indigenous Tweets: The Reo Māori Twitter Corpus**
*Language Resources and Evaluation*

David Trye, Te Taka Keegan, Paora Mato and Mark Apperley
University of Waikato, New Zealand
Corresponding Author: [dtrye@waikato.ac.nz](mailto:dtrye@waikato.ac.nz)

**Online Resource 1: Metadata in the RMT Corpus**

| **Variable Name** | **Data Type** | **Description** |
| --- | --- | --- |
| id | - | Twitter's unique identifier for the tweet. |
| content | Text | The tweet text with consistent formatting applied to user mentions and links. Special characters have been eliminated. |
| content_with_emojis | Text | The tweet text with consistent formatting applied to user mentions and links. Special characters have been preserved. |
| conversation_id | Categorical | The ID of whichever tweet initiated the conversation. |
| in_reply_to_user_id | Categorical | If the tweet is written in reply to another, this is the ID of the user who wrote the original tweet. |
| date | Temporal | The timestamp when the tweet was posted. |
| error | Categorical | The reason why the tweet could not be downloaded, if there was an error ('Authorization Error', 'Not Found Error', 'None'). |
| favourites | Quantitative | The number of favourites (likes, retweets & quotes) that the tweet received. |
| like_count | Quantitative | The number of likes that the tweet received. |
| quote_count | Quantitative | The number of times that the tweet was quoted. |
| reply_count | Quantitative | The number of replies that the given tweet received. |
| retweet_count | Quantitative | The number of retweets that the tweet received. |
| lang | Categorical | The two-letter code representing the language that the tweet was (erroneously) classified as (never Māori as the Twitter API does not have official support for te reo). |
| media | - | Links to any photos or videos featured in the tweet. |
| outlinks | - | Links to any external sites mentioned in the tweet. |
| source_label | Categorical | The device or third-party application from which the tweet was sent. |
| url | - | The link for viewing the tweet in context on Twitter. |
| year | Quantitative | The year the tweet was written (between 2007 and 2020). |
| maori_words | Text | The list of Māori words detected in the tweet. |
| num_maori_words | Quantitative | The number of Māori words detected in the tweet (i.e. the number of items in *maori_words*) |
| percent_maori | Quantitative | The percentage of Māori text detected in the tweet (=num_maori_words / total_words * 100). |
| total_words | Quantitative | The total number of words in the tweet. |
| user.id | - | Twitter's unique identifier for the user who wrote the tweet. |
| user.username | - | The username of the account from which the tweet was posted. |
| user.alias | - | An alias for the author of the tweet in the form T<X>, where <X> represents the user’s ranking based on their total number of tweets in the corpus (user.num_tweets). |
| user.display_name | - | The user’s display name on Twitter. |
| user.location | Text | The (unedited) location of the user. |
| user.region | Categorical | The user's location, based on self-reported information. Where possible, the data has been aggregated into New Zealand regions and names of overseas countries. |
| user.gender | Categorical | The user’s gender if the account represents an individual (‘male’, ‘female’, ‘gender-neutral’, ‘unknown’) or ‘group’ if the account represents multiple people. |
| user.ethnicity | Categorical | The user’s ethnicity, extracted from the account description (user.description). |
| user.iwi | Categorical | The user’s tribal affiliation(s) if they are of Māori descent. |
| user.created | Temporal | The date on which the user’s account was created. |
| user.description | Text | The user’s self-written account description (bio). |
| user.description_urls | - | Any links mentioned in the user’s account description (user.description). |
| user.status | Categorical | The account status (as of December 2020) of the user who wrote the tweet: 'active', 'protected', 'suspended' or 'not found'. |
| user.favourites_count | Quantitative | The total number of favourites that the user has received (not just counting tweets in the RMT Corpus). |
| user.followers_count | Quantitative | The user's number of followers (as of December 2020). |
| user.friends_count | Quantitative | The number of accounts that the user follows (as of December 2020). |
| user.link | - | The link for viewing the user’s profile on Twitter. |
| user.link_tcourl | - | The short version of any links associated with the user’s profile (separate from their account description). |
| user.link_url | - | Any links associated with the user’s profile (separate from their account description). |
| user.listed_count | Quantitative | The number of people who have tagged the user in one or more tweets (not just counting tweets in the RMT Corpus). |
| user.media_count | Quantitative | The number of tweets posted by the user that have included media items (not just counting tweets in the RMT Corpus). |
| user.num_tweets | Quantitative | The total number of tweets in the RMT Corpus that were written by this user. |
| user.prof_banner_url | - | The link to the user’s profile banner. |
| user.prof_image_url | - | The link to the user’s profile picture. |
| user.statuses_count | Quantitative | The total number of statuses that the user has posted (not just counting tweets in the RMT Corpus). |
| user.verified | Categorical | Whether or not the user’s account is verified (‘true’ or ‘false’). |
